# Prey Capture, Ingestion, and Digestion Dynamics of *Octopus vulgaris* Paralarvae Fed Live Zooplankton

**DOI:** 10.3389/fphys.2017.00573

**Published:** 2017-08-17

**Authors:** Manuel Nande, Pablo Presa, Álvaro Roura, Paul L. R. Andrews, Montse Pérez

**Affiliations:** ^1^Laboratory of Marine Genetic Resources, Faculty of Biology, University of Vigo Vigo, Spain; ^2^Grupo de Acuicultura Marina, IEO-Vigo Vigo, Spain; ^3^ECOBIOMAR, Instituto de Investigaciones Marinas, Consejo Superior de Investigaciones Científicas Vigo, Spain; ^4^Department of Biology and Evolution of Marine Organisms, Stazione Zoologica Anton Dohrn Napoli, Italy

**Keywords:** digestion dynamics, digestive tract motility, nutrition physiology, *Octopus vulgaris* paralarvae, predatory behavior, video analysis, zooplankton

## Abstract

*Octopus vulgaris* is a species of great interest in research areas such as neurobiology, ethology, and ecology but also a candidate species for aquaculture as a food resource and for alleviating the fishing pressure on its wild populations. This study aimed to characterize the predatory behavior of *O. vulgaris* paralarvae and to quantify their digestive activity. Those processes were affordable using the video-recording analysis of 3 days post-hatching (dph), mantle-transparent paralarvae feeding on 18 types of live zooplanktonic prey. We show for the first time in a live cephalopod that octopus paralarvae attack, immobilize, drill, and ingest live cladocerans and copepods with 100% efficiency, which decreases dramatically to 60% on decapod prey (*Pisidia longicornis*). The majority (85%) of successful attacks targeted the prey cephalothorax while unsuccessful attacks either targeted the dorsal cephalothorax or involved prey defensive strategies (e.g., juvenile crab megalopae) or prey protected by thick carapaces (e.g., gammaridae amphipods). After immobilization, the beak, the buccal mass and the radula were involved in exoskeleton penetration and content ingestion. Ingestion time of prey content was rapid for copepods and cladocerans (73.13 ± 23.34 s) but much slower for decapod zoeae and euphausiids (152.49 ± 29.40 s). Total contact time with prey was always <5 min. Contrary to the conventional view of crop filling dynamics observed in adult *O. vulgaris*, food accumulated first in the stomach of paralarvae and the crop filled after the stomach volume plateaued. Peristaltic crop contractions (~18/min) moved food into the stomach (contractions ~30/min) from where it passed to the caecum. Pigmented food particles were seen to enter the digestive gland, 312 ± 32 s after the crop reached its maximum volume. Digestive tract contents passed into the terminal intestine by peristalsis (contraction frequency ~50/min) and defaecation was accompanied by an increased frequency of mantle contractions. Current results provide novel insights into both, *O. vulgaris* paralarvae—live prey capture strategies and the physiological mechanisms following ingestion, providing key information required to develop an effective rearing protocol for *O. vulgaris* paralarvae.

## Introduction

*Octopus vulgaris* is the best known octopod species among Octopodidae (Norman et al., 2014) and one of the most intensively studied species in various animal research areas such as development and growth (e.g., Villanueva and Norman, 2008; Iglesias and Fuentes, 2014), behavior (e.g., Hanlon and Messenger, 1996; Fiorito and Gherardi, 1999), and neuroscience (for a review see Fiorito et al., 2014). Particularly interesting is the well-developed central nervous system in *O. vulgaris* which makes it a suitable model organism in neurophysiology, ethology, and ecology (for reviews see Wells, 1978; Hanlon and Messenger, 1996; Hochner et al., 2006; Hochner, 2012; Fiorito et al., 2014). Additionally, *O. vulgaris* has been a candidate for aquaculture since Classical Antiquity (Iglesias et al., 2007; Lotze et al., 2011) and such interest continues stimulated by concerns about its sustainability despite the large size of the commercial cephalopod fishery (Iglesias et al., 2007; Vidal et al., 2014; Doubleday et al., 2016).

Massive mortality during the paralarval stage is one of the major bottlenecks to the successful rearing of the common octopus. Such mortality is believed to be caused by our deficient knowledge of early nutritional requirements (Iglesias and Fuentes, 2014; Navarro et al., 2014; Vidal et al., 2014). For instance, prey attack strategies, types of live zooplanktonic prey preferred and the physiology of digestion, are essentials to ensure survival during early developmental stages in the hatchery. The zootechnical advances in paralarvae growth will also facilitate the provision of captive bred animals for a variety of research studies. The latter may become important as European Union Directive 2010/63/EU (European Parliament Council of the European Union, 2010) prohibits the use of animals taken from the wild unless this can be scientifically justified (*Article* 9).

Wild octopus paralarvae are believed to feed on a large number of zooplankton species, some of which have been identified with molecular tools (Roura et al., 2012). Different types of crab zoeae (Villanueva, 1995; Iglesias et al., 2004), copepod prey (Iglesias et al., 2007), and wild zooplankton (Estévez et al., 2009) have been assayed in nutritional trials of common octopus paralarvae and have improved its early growth and survival. Also, adapted live prey diets based on gammarid amphipods have improved growth and survival of benthic octopuses such as *Octopus joubini* and *Octopus maya* (Forsythe and Hanlon, 1980; Baeza-Rojano et al., 2013).

Understanding the nutritional gain achievable using live prey requires the design of parallel studies to properly dissect the different phases of the octopus attack strategy and ingestion dynamics. Feeding strategies have been described in wild adults as well as in captive animals (for review see Wells, 1978; Hanlon and Messenger, 1996).

**Figure 1:**
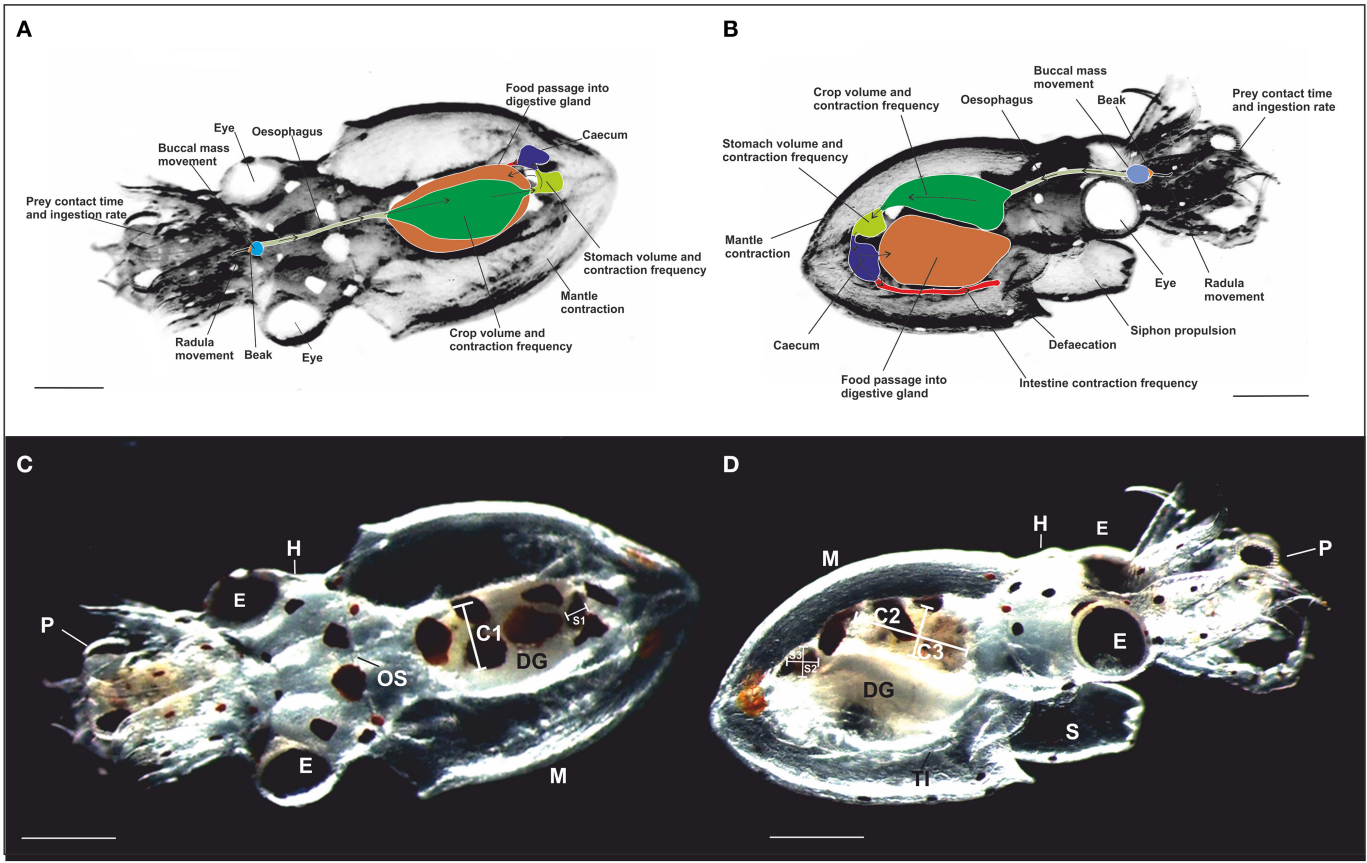
Schematic representation of the dorsal **(A)** and the ventral **(B)** view of a paralarva in contact with prey (crab zoea) showing the location of the main digestive tract structures measured. Arrows indicate the direction of the food passage. **(C,D)** photography of the dorsal view **(A)** and the ventral view **(B)** taken from video recordings of a 3 dph free-swimming paralarvae of *O. vulgaris* with crab zoea prey captured. Note the transparency of the paralarva enabling direct observation of the digestive tract. The diameter of the crop **(C)** and the stomach (S) was measured along three axes: C1 and S1 (medio-lateral width), C2 and S2 (rostro-caudal length), and C3 and S3 (dorso-ventral thickness). OS, Oesophagus; DG, digestive gland; TI, terminal part of the intestine; E, eye; H, head; P, prey; M, mantle; and S, siphon. Scale bar = 0.5 mm.

The buccal mass is the most anterior part of the cephalopod digestive tract and all its components are already present at hatching (Villanueva and Norman, 2008). The buccal mass comprises two chitinous beaks, the radula and the associated musculature (Figures 1A,B; Altman and Nixon, 1970; Boucher-Rodoni, 1973; Boyle et al., 1979a,b; Guerra and Nixon, 1987) and can be rotated by the buccal musculature under neural control (Altman and Nixon, 1970; Boyle et al., 1979a,b). Adults use their beak to bite or to drill the prey exoskeleton thus creating an access to inject digestive enzymes from the posterior salivary glands into the prey via the salivary papilla (Wells, 1978). The radula in adults is equipped with an erect part with small teeth which are used to rasp food into the mouth (Nixon, 1968). Such food ingestion has also been reported in paralarvae which fully ingested crab zoeae fluids leaving an empty exoskeleton (Hernández-García et al., 2000). In adults, food passes through the esophagus to the crop, proceeds into the stomach and caecum, and transits to the digestive gland for nutrient absorption (Boucaud-Camou et al., 1976; Boucaud-Camou and Boucher-Rodoni, 1983; O'dor et al., 1984; Linares et al., 2015). The contractile activity of esophagus, crop, stomach, caecum, and intestine, progressively moves the food along the digestive tract (Figures 1A,B), i.e., a complex physiological activity believed to be coordinated by the gastric ganglion, although additional hormonal control cannot be excluded (Andrews and Tansey, 1983).

There is a paucity of knowledge of feeding strategies and digestive tract physiology of paralarvae as compared to adult octopus. To date, only indirect assessments of prey consumption have been possible as inferred from molecular analysis of paralarvae (Roura et al., 2012, 2016). In fact, direct assessment of feeding in recently hatched octopus paralarvae is elusive and requires the study of prey-predator relationships (e.g., hunting, defense, and escape) as well as of the subsequent prey capture, ingestion and post-ingestion. One way to access such knowledge is to quantify behavioral and physiological data from high-resolution video recording (e.g., Fiorito and Scotto, 1992). Video recording allows the simultaneous quantification of multiple physiological processes on the same animal. Moreover, the transparency of the thin mantle muscles of cephalopod paralarvae (except for sparse chromatophores) allows real time video monitoring and the quantification of the digestive process in a live, non-invasive manner and therefore, to undertake a comprehensive investigation of octopus paralarvae feeding in captivity (Hernández-García et al., 2000).

The general aim of this study is to understand the feeding strategies employed by common octopus paralarvae on different prey types and the physiological mechanisms operating during their digestion. The specific objectives were the *in vivo* quantification of (a) the attack strategy and related behavior exhibited by octopus paralarvae fed wild zooplankton, spider crab zoeae and edible crab zoeae hatched from broodstock, (b) the dynamics of exoskeleton penetration (“drilling”) and content ingestion of different prey types, and (c) the dynamics of food distribution in the crop and the stomach comprising the motility changes occurring throughout the digestive tract until defaecation.

## Materials and methods

### Biological material

#### Octopus broodstock

In January and February, from 2013 to 2016, adult female and male *O. vulgaris*, Cuvier 1797, were captured in the Ría de Vigo (NW Spain) using artisanal fishing gear. Fourteen individuals were transported to the aquaculture facilities of *Centro Oceanográfico de Vigo* (COV-IEO) using portable—100 L tanks at 14°C and O_2_ saturation. Transport lasted 20 min and the broodstock was maintained in a flow-through concrete tank (4.60 × 2.10 m) filled with seawater (1.0 m in depth) at 14–18°C and 35 psu (practical salinity unit) as measured weekly using a refractometer ATC (ATAGO^©^; Iglesias et al., 2016). Several sections of a plastic pipe (0.2 m in diameter and 0.5 m in length) were immersed in the tanks as dens providing shelter for spawning females. The female to male ratio was 3:1 and the broodstock was fed frozen mussels (*Mytilus galloprovincialis*), frozen fish (*Merluccius merluccius* and *Sardina pilchardus*), and frozen crustaceans (*Polybius* spp.) three times a week. The food rations were calculated as 20% of the broodstock biomass introduced into each tank. The spawning females were removed from the broodstock tank and housed individually in smaller tanks (1.0 × 1.0 m) filled with seawater, 1.0 m depth at the ambient temperature of Ría de Vigo (14–18°C). Ammonia, nitrites and nitrates were measured daily and kept close to zero (Nutrafin^©^). Dissolved oxygen was measured twice a day (early morning and late afternoon) using an oximeter (OxyGuard-10XHM053, Polaris^©^, UK) and always maintained above 90%. Females spawned for 8–9 days between March and June each year (2013–2016) and laid their egg batches on the upper side of the pipe, so egg clusters remained suspended allowing their constant cleaning and oxygenation by gentle water jets from the female's siphon.

#### Hatching paralarvae

The embryonic development of *O. vulgaris* paralarvae lasted 45–65 days at a seawater temperature of 14–18°C (Nande et al., 2017). Paralarvae remained in the hatching tank until day 2 post hatching (dph) and were transferred thereafter to 5 L buckets with filtered seawater (0.1 μm) at 35 psu salinity and 18°C for 24 h before carrying out the experiments. Thirty-five, 3 dph paralarvae per bucket (already devoid of inner yolk to prevent interference with their feeding behavior) were used in the experiments (Nande et al., 2017). Paralarvae were anesthetized at the end of the study by immersing them in a 0.5% MgCl_2_ seawater solution (Magnesium chloride hexahydrate, Barcelonesa^©^, Global Chemical Solutions, Barcelona, Spain) at room temperature (18–20°C) for 10 min and was increased to 3.5% for 30 min. All paralarvae were killed by destruction of the brain with a needle and aided by binocular microscope (LEICA MZ8®). Although, current experiments do not fall under Directive 2010/63/EU (European Parliament Council of the European Union, 2010) the authors followed its principles in terms of minimizing the number of animals used (Fiorito et al., 2014) and by using an appropriate killing method (Andrews et al., 2013; Fiorito et al., 2015). All the experimental procedures were supervised by an ethics committee (Octowelf project, see below, #CEIBA 2014-0108).

#### Zooplankton samples

Six marine surveys were conducted between 2013 and 2016 aboard the Oceanographic vessel “José María Navaz” at different sampling points of Ría de Vigo. Three trawls were performed per survey using a planktonic net of 2 m in diameter with a 500 μm mesh placed in the collector tip and dragged to an average depth of 10 m for 10 min. Zooplankton samples were filtered twice through a 2 mm sieve and maintained in 100 L tanks containing seawater and equipped with gentle aeration. Each zooplanktonic sample was transferred and maintained in a specific 500 L tank equipped with gentle aeration and constant temperature (18°C) until completion of each feeding experiment (2 days).

#### Crustacean broodstock

Several broodstocks of the crabs *Cancer pagurus* and *Maja brachydactila* were reared between 2012 and 2016 at the COV-IEO facilities to obtain live zoeae for nutritional assays on *O. vulgaris* paralarvae. Female crabs were acclimatized and maintained at the ambient temperature of Ría de Vigo (14–18°C) and at low light intensity (<100 lx) in 1.0 × 1.0 m tanks of 0.75 m in depth, supplied with filtered seawater in a flow through system. Crabs were fed frozen mussels (*M. galloprovincialis*) three times a week at a ratio of 10% in weight of the crab broodstock biomass. Spontaneous hatching events of zoeae were collected in spring and transferred to 100 L tanks using a 500 μm sieve. The water temperature was maintained at 18°C in a recirculating closed circuit.

### Experimental design

#### Effective attacks vs. ineffective attacks

*O. vulgaris* paralarvae previously acclimatized in 5 L buckets (*n* = 35 paralarvae/bucket) filled with 0.1 μm filtered seawater of 35 psu at 18°C and kept in low light intensity (100–300 lx) were used in the experiments. Prey density in each bucket was 0.2 individuals/mL and a gentle air-flow was used to mix the water and facilitate prey-paralarvae encounters. This procedure was repeated so that in total three buckets per sampling, each with 35 paralarvae were studied. Only the 18 types of prey captured by paralarvae were identified under a binocular microscope (LEICA MZ8®) as helped by taxonomic identification guides (Rose, 1933; Trégouboff and Rose, 1957). Taxonomic identification reached either the species or the family level (Table 1). After 10 min of paralarvae-prey co-habitation in buckets, 15 paralarva-prey combinations (PPC) were carefully collected per replicate using a Pasteur pipette and examined under a binocular microscope (LEICA MZ8®). The number of paralarvae–prey combinations (PPC), the number of “effective attacks“(EA) and the number of “ineffective attacks” (IA) were defined as follows,

- Paralarvae-prey combination (PPC): a sustained predator-prey association, i.e., the prey is immobilized within the paralarva's arms.
- Effective attacks (EA): the paralarva grasps the prey, pierces its exoskeleton, and ingests its body content.
- Ineffective attacks (IA): the paralarva grasps the prey but neither pierces it nor ingests its content.

**Table 1:** Classification of wild zooplankton and laboratory bred species (^*^) captured by octopus paralarvae.

**Type of prey**	***N* Total**	**EA (%)**	**IA (%)**	**L (mm)**	**W (mm)**
*Acartia clausii*	13.67 ± 1.53	100	0	1.25 ± 0.11	0.25 ± 0.01
*Temora longicornis*	15.00 ± 2.64	100	0	1.57 ± 0.12	0.61 ± 0.01
*Centropages* sp.	15.00 ± 1.73	100	0	1.98 ± 0.13	0.57 ± 0.01
*Podon intermedius*	15.34 ± 1.53	100	0	0.90 ± 0.01	0.45 ± 0.01
*Carcinus maenas* zoeae	12.67 ± 3.06	80.00 ± 8.66	20.00 ± 8.66	1.73 ± 0.15	0.58 ± 0.01
*Maja brachydactyla* zoeae^*^	17.00 ± 2.65	86.39 ± 2.27	13.61 ± 1.27	2.14 ± 0.05	0.69 ± 0.01
*Cancer pagurus* zoeae^*^	14.00 ± 3.00	83.30 ± 2.11	16.70 ± 2.11	2.45 ± 0.07	0.71 ± 0.02
*Pisidia longicornis*	09.00 ± 2.00	62.48 ± 4.87	37.52 ± 4.87	1.47 ± 0.02	0.57 ± 0.01
Paguridae^a^	10.34 ± 3.21	72.95 ± 9.06	27.05 ± 9.06	2.19 ± 0.15	0.86 ± 0.02
Processidae^a^	13.00 ± 2.00	68.73 ± 4.87	31.27 ± 4.87	4.34 ± 0.24	0.65 ± 0.01
Hippolytidae^a^	11.34 ± 4.51	63.77 ± 7.59	36.23 ± 7.59	2.35 ± 0.13	0.75 ± 0.04
Palaemonidae^a^	12.00 ± 2.00	74.52 ± 4.31	25.48 ± 4.31	3.34 ± 0.21	0.73 ± 0.03
*Nyctiphanies couchii*	13.00 ± 1.00	71.49 ± 6.27	28.51 ± 6.27	5.46 ± 0.34	0.83 ± 0.03
Crangonidae^a^	07.00 ± 1.00	0	100	4.88 ± 0.23	0.88 ± 0.03
Gastropods^a^	03.67 ± 0.58	0	100	0.57 ± 0.01	0.50 ± 0.01
Gammaridae^a^	06.34 ± 1.15	0	100	2.46 ± 0.14	1.45 ± 0.06
Hyperiid amphipod^a^	05.34 ± 1.15	0	100	1.99 ± 0.10	1.15 ± 0.09
Brachyura megalopae^a^	03.67 ± 0.58	0	100	1.68 ± 0.01	2.34 ± 0.11

a *A finer taxonomic classification of early stages could not be achieved in this taxon*.

*N Total is the mean and standard deviation of the No. of prey captured by paralarvae from 2013 to 2016. EA and IA are the mean percentages (±sd) of effective attacks and ineffective attacks per prey type, respectively. The length (L) and the width (W) of prey are given in millimeters*.

#### Dynamics of food ingestion and digestive passage

Paralarvae-prey combinations (PPC) were photographed and filmed in a Petri dish filled with seawater, using a high-resolution camera (Leica IC80 HD®, 3.1 Mpx) mounted on a binocular microscope (LEICA MZ8®). Recording of food passage through the digestive tract of paralarvae following EA was possible due to the transparency of the mantle musculature. The duration of recordings varied depending on the paralarva—prey association time and the orientation of the paralarva, so the observation time of digestive activities in crop, stomach, and terminal intestine differed between specimens. For those reasons, most parameterization is given as number (No.) of events/10 s rather than as No. events/min. Maximum stomach dimensions (diameter and radius) were estimated using image analysis software (LEICA Application Suite V4®) once the meal stopped moving into the stomach and began accumulating in the crop. Crop dimensions were taken once paralarvae released the prey carcass. Those dimensions were used to calculate the crop volume and the stomach volume, respectively.

Volume of the crop (Cv) and volume of the stomach (Sv) were calculated according to the formula,

$$\begin{array}{l} Cv\;or\;Sv=\;\frac{4}{3\pi }\;\cdot \;r1\cdot r2\cdot r3 \end{array}$$

Diameters of the stomach and the crop were measured from videotape recordings along three axes: as X (rostro-caudal length), Y (medio-lateral width), and Z (dorso-ventral thickness). Diameters of the stomach and the crop were used to calculate their radius per axis (r1, r2, r3) and were applied in the above formula to calculate the volume at specific time points during EA (Figures 1C,D).

After some preliminary observations for calibration, the association between paralarvae and prey was split into three phases as follows,

1. Initial phase (IP): the time since paralarvae captured the prey until ingestion began.
2. Middle phase (MP): the time since the food stopped entering the stomach until it began to accumulate in the crop.
3. Late phase (LP): the time since either the crop was full or since paralarvae released the prey.

Videos of PPC involving 18 prey types as well as single paralarvae were reanalyzed to measure the following parameters over 10 s intervals during the three defined phases (IP, MP, LP),

- Mantle contractions (MC) during PPC.
- Siphon propulsions (SP), measured as sudden movements provoked by siphon jetting during the fight between prey and paralarvae (PPC).
- Buccal mass movement (BM) during different phases (IP, MP, and LP).
- Radula movement (RM) during prey penetration and ingestion.
- Stomach contraction frequency (SC), volume (v), and nature of the stomach contents from each prey type.
- Peristaltic crop movements (PC) propelling food toward the stomach. The crop volume was measured during LP.
- Ingestion rate (IR), estimated as,

$$\begin{array}{l} \text{IR}=\frac{TF}{Ti} \end{array}$$

Where *TF* is the total volume of food ingested (crop volume + stomach volume when the paralarvae released the prey) and *Ti* is the total ingestion time employed by paralarvae.

h) Contractions of the terminal intestine (TIC) measured in all phases of the ingestion (IP, MP, and LP).
i) Total food intake (TF) was the sum of the stomach volume (Sv) and the crop volume (Cv) as defined above.
j) Ingestion time (IT) was the time from the start of ingestion (food passing through the esophagus) until the prey is released.
k) Total contact time (TCT), is defined as the time from prey capture until release.
l) Contraction frequency of the mantle during post-ingestion.
m) Peristaltic crop movement and periods of crop inactivity (CI) during post-ingestion. CI measures the time from when the peristaltic activity (PC) ceases until the beginning of the next PC episode in the crop.
n) Description of food passage thought the intestine tract, caecum, digestive gland, excretion, and terminal intestine contraction (TIC) during post-ingestion.

Likewise, three phases were defined for ineffective attacks (IA) and used in video analyses,

1. Initial phase (IP): the period prior to prey capture.
2. Middle phase (MP): the period between prey capture and its immobilization (fight initiation).
3. Late phase (LP): the period from prey immobilization to prey release.

### Statistics

Predator-prey data measured from video recordings were tested for homoscedasticity using the Levene's test (Zar, 1999). Data distributions were checked for normality using the one-sample Kolmogorov–Smirnoff test (Zar, 1999). Kruskal–Wallis analysis was used to evaluate the significance of nonparametric data from EA and IA relative to the different prey types. Differences in the mean of all parameters (Mantle contraction, MC; buccal mass movement, BM; radula movement, RM; intestine contraction, TIC; crop volume, Cv; stomach volume, Sv; total time of ingestion, TIT; and ingestion rate, IR) among replicates and among, prey type, were compared with one-way ANOVA using the program STATISTICA 10.0^©^. Global significant tests led to comparison of pairwise means using the Tukey test. Intra-individual analyses of the same variable taken at different digestion phases (IP, MP, and LP) over time were performed with a *t*-test and a general linear model analysis of variance (ANOVA-RM) for repeated measures. Data are presented in the text and illustrations as mean ± *SD* and significant differences were assumed below the nominal probability threshold *p* = 0.05.

## Results

### Paralarvae attack and prey capture

**Figure 2:**
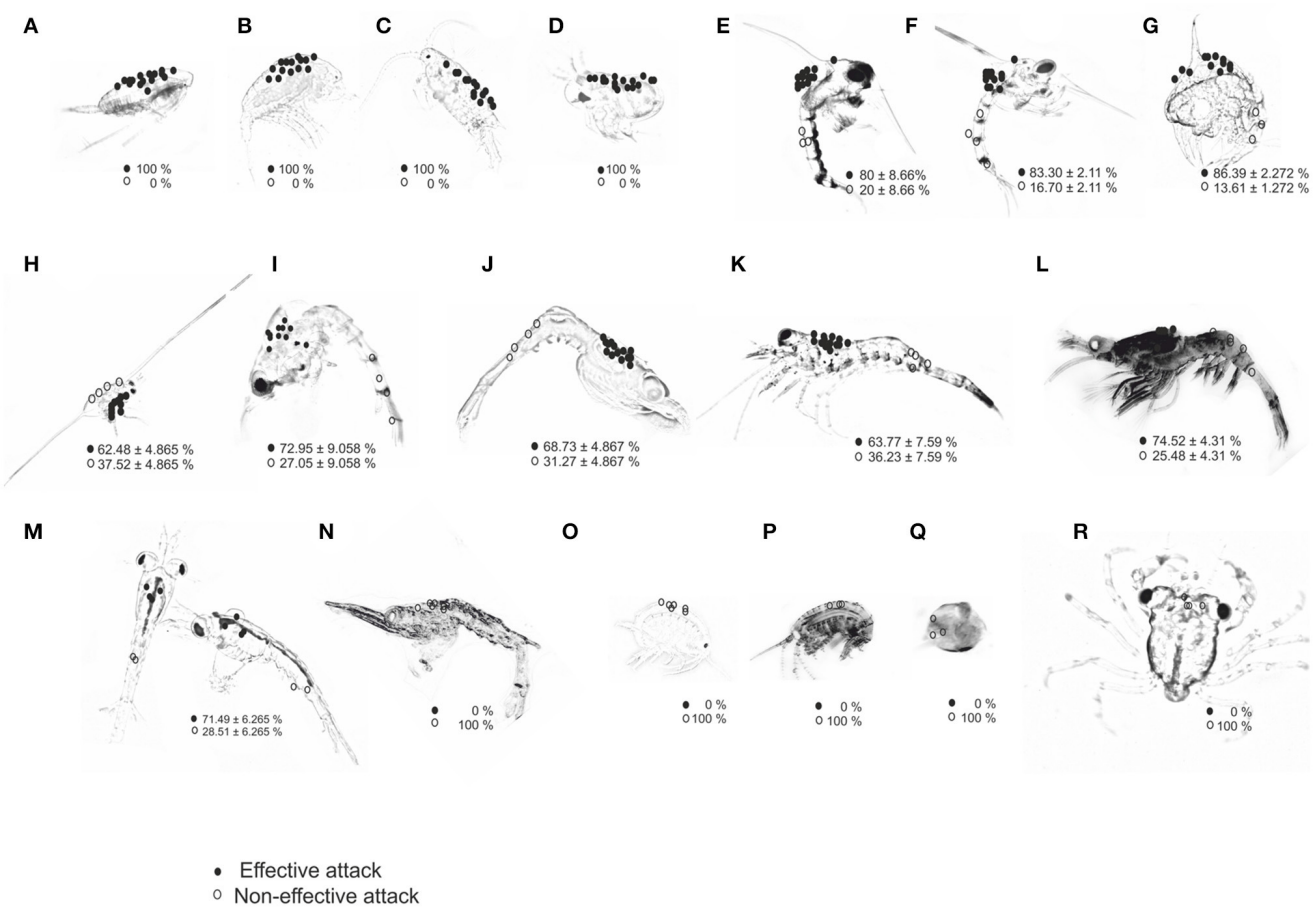
Location of attack sites (circles) by paralarvae of *O. vulgaris* on different prey types: **(A)** *Acartia clausii*; **(B)** *Temora longicornis*; **(C)** *Centropages* sp.; **(D)** *Podon intermedius*; **(E)** *Carcinus maenas*; **(F)** *Cancer pagurus*; **(G)** *Maja brachydactyla*; **(H)** *Pisidia longicornis*; **(I)** Paguridae; **(J)** Processidae; **(K)** Hippolytidae; **(L)** Palaemonidae; **(M)** *Nyctiphanes couchii*; **(N)** Crangonidae; **(O)** Gammarid amphipod; **(P)** Hyperiid amphipod; **(Q)** Gastropods; **(R)** Brachyura megalopae. Prey images are not to scale to identify all preys in the same panel; actual prey size is given in Table 1. Percentage of EA (effective attacks, filled circles) and IA (ineffective attacks, open circles) are given per prey type.

Paralarvae exposed to wild zooplankton captured diverse prey including cladocerans (*Podon intermedius*), copepods (*Acartia clausii, Temora longicornis*, and *Centropages* sp.), zoeae of *Carcinus maenas* and *Pisidia longicornis* as well as zoeae of the decapod families Crangonidae, Hippolytidae, Paguridae, Palaemonidae, and Processidae (Table 1). Paralarvae also captured larval stages of the euphausiids *Nyctiphanes couchii*, amphipods (e.g., gammarids and hyperiids) and megalopae stages of gastropods and crabs. Prey capture was independent of prey size or prey mobility in the water column (Figure 2). The defensive strategy of prey encountering paralarvae varied substantially between species. Copepods showed continuous swimming and sudden direction changes; zoeae (*C. maenas, C. pagurus*, and *Maja brachydactyla*) showed rhythmic swimming, increased frequency of abdominal flexion and high speed spinning (Video, from start to second 17; Supplementary Material). Megalopae used their chelipeds as a defensive tool (Video, from second 17 to second 33; Supplementary Material). The sharp spines of zoeae occasionally damaged or even killed paralarvae (Video, from second 33 to second 43; Supplementary Material).

### Prey capture frequency, prey target site, and attack effectiveness

The dorsal-cephalothorax was the site where 100% of effective attacks (EA) occurred on small prey (copepods and cladocerans), crab zoeae, and krill (Figure 2; Table 1). The frequency of efficient attacks (EA) on *M. brachydactyla* averaged 86.4 ± 2.3% but was less on Hippolytidae and *P. longicornis* (≈60%, Table 1). In laboratory-bred strains and in wild decapod species, EA always targeted the cephalothoracic area, occurring 85.0 ± 5.0% on its dorsal area and 10.0 ± 5.0% on its lateral or ventral areas (Figure 2; Video, from second 44 to second 60, Supplementary Material). All ineffective attacks (IA) on laboratory bred decapod zoeae and on wild zooplankton taxa (*C. maenas, P. longicornis*, Paguridae, Processidae, Hippolytidae, Palaemonidae, and the krill *N. couchii*) occurred on the abdominal or on the telson areas (Figure 2; Table 1).

Significant differences in the frequency of EA were observed between prey types grouped by categories: (a) copepods and cladocerans (100% EA), (b) decapod zoeae and krill (60–90% EA), and (c) Crangonidae, amphipods, gastropods, and crab megalopae (0% EA) [Kruskal–Wallis test, *H* (18 prey types, *n* = 57) = 51.02; *p* = 0.0001]. All captures of decapod zoeae from families Crangonidae, amphipods, gastropods, and crab megalopae occurred on the dorsal cephalothorax and resulted in ineffective attacks (IA) (paralarvae were unable to drill their exoskeleton). All attacks on crab megalopa occurred on the dorso-anterior region and prey employed their chelipeds to successfully defend from paralarvae.

### Prey killing and ingestion

#### Effective attacks and initial digestive tract activity

During the initial phase (IP) of the predator-prey combination (PPC), paralarvae fought with the prey to immobilize it (Video, from second 44 to second 60, Supplementary Material).

**Figure 3:**
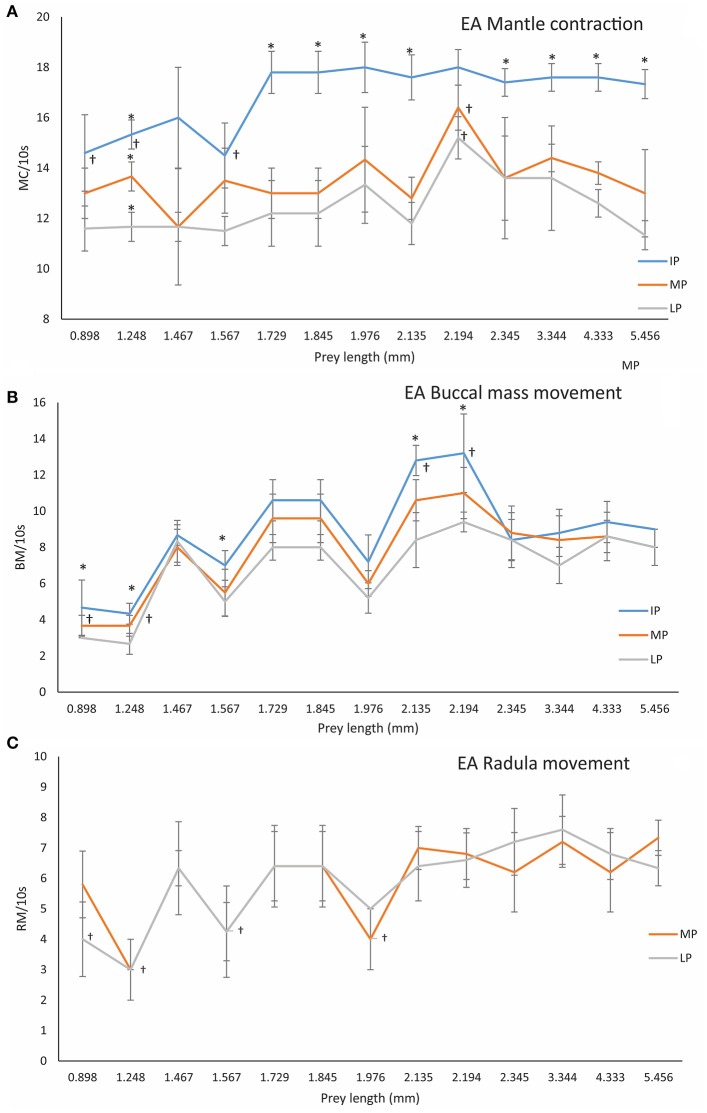
Frequency of MC (Mantle contractions, **A**), BC (Buccal mass movements, **B**) and RM (Radula movements, **C**), in different PPC (predator-prey combination phases), i.e., IP (initial phase), MP (middle phase), and LP (late phase) of EA (effective attacks) by 3 dph *O. vulgaris* paralarvae in relation to prey length. Data are plotted as mean ± *SD*. Symbols (^†^) indicate significant differences between prey-lengths in the same phase (one-way ANOVA, *p* < 0.05). The asterisk (^*^) indicates significant differences within prey-type between PPC phases (ANOVA-RM, *p* < 0.05; see Section Materials and Methods for details).

The frequency of MC (mantle contractions) in the IP, calculated as an average from all EA was 17.08 ± 1.44/10 s (*n* = 66). The MC frequency was significantly less in small prey (*A. clausii, T. longicornis*, and *P. intermedius*) than in large prey (Paguridae, Processidae, Hippolytidae, Palaemonidae, and the krill *N. couchii*) in all phases, i.e., IP (ANOVA-RM, *F* = 43.34, *p* = 0.0001), MP (ANOVA-RM, *F* = 189.23, *p* = 0.00001), and LP (ANOVA-RM, *F* = 176.23 *p* = 0.0001; Figure 3A; Table 2). Mantle contractions were accompanied by siphon propulsions averaging SP = 3.03 ± 0.54/10 s (Table 1 in Supplementary Material), as well as by an average of 12 movements/min of repetitive lateral movements of head and arms. The initiation of the buccal mass activity was coincident with movements of siphon, head and arms (Video, from second 44 to second 60, Supplementary Material). The MC frequency during phase MP (MC = 13.67 ± 1.43/10 s) and phase LP (MC = 12.73 ± 1.76/10 s) were significantly less than the MC frequency during phase IP (MC = 17.08 ± 1.44/10 s) (ANOVA-RM, *n* = 66, *F* = 201.46, *p* = 0.0001; Figure 3A).

**Table 2:** Mean frequency and standard deviation of mantle contractions, crop peristaltic contractions, intervals between episodes of crop activity, and terminal intestine contractions during the post-ingestion, i.e., after paralarvae released prey.

**Type of prey**	**No. paralarvae**	**No. Mantle Contractions /10 s**	**No. Crop contractions/10 s**	**Interval between episodes of crop peristaltic movements/10 s**	**No. terminal intestine contractions/10 s**
Copepods *(A. clausii, T. longicornis, Centropages* sp.)	3	5.667 ± 0.577	3.333 ± 0.254	6.667 ± 0.334	8.200 ± 0.545
Cladocera (*Podon intermedius*)	4	6.334 ± 0.577	2.330 ± 0.667	6.167 ± 0.687	8.333 ± 1.334
Zoeae *M. brachydactyla, C. pagurus*	6	6.334 ± 1.154	3.600 ± 0.667	6.334 ± 0.236	9.600 ± 0.547
Zoeae *C. maenas, P. longicornis*	6	6.000 ± 1.000	3.200 ± 0.767	6.867 ± 0.381	9.200 ± 0.667
Paguridae	3	6.333 ± 1.155	2.800 ± 0.476	7.000 ± 1.000	8.600 ± 0.845
Processidae, Hippolytidae, Palaemonidae	8	6.667 ± 0.577	3.333 ± 0.564	6.670 ± 0.334	9.333 ± 0.577
Euphausiidae	4	5 ± 1.732	2.800 ± 0.776	5.600 ± 0.658	8.800 ± 0.955

*Data are grouped according to the criterion of prey group (see text for definitions and details)*.

Once the prey was immobilized, paralarvae prepared to access its internal tissues using the beak and buccal mass movements (BM). In IA (ineffective attacks), the frequency of BM was 6.16 ± 2.64/10 s for copepods and cladocerans as compared to 9.24 ± 1.29/10 s for the rest of the species. BM movement was significantly higher on larger prey than on smaller prey (e.g., copepods and cladocerans) (one-way ANOVA, *F* = 29.064, *p* = 0.00001; Figure 3B). Significant differences in BM were also observed between phases IP and LP (e.g., BM_IP = 9.08 ± 2.58/10 s vs. BM_LP = 7.24 ± 2.05/10 s; ANOVA-RM, *F* = 112.23, *p* = 0.0001).

**Figure 4:**
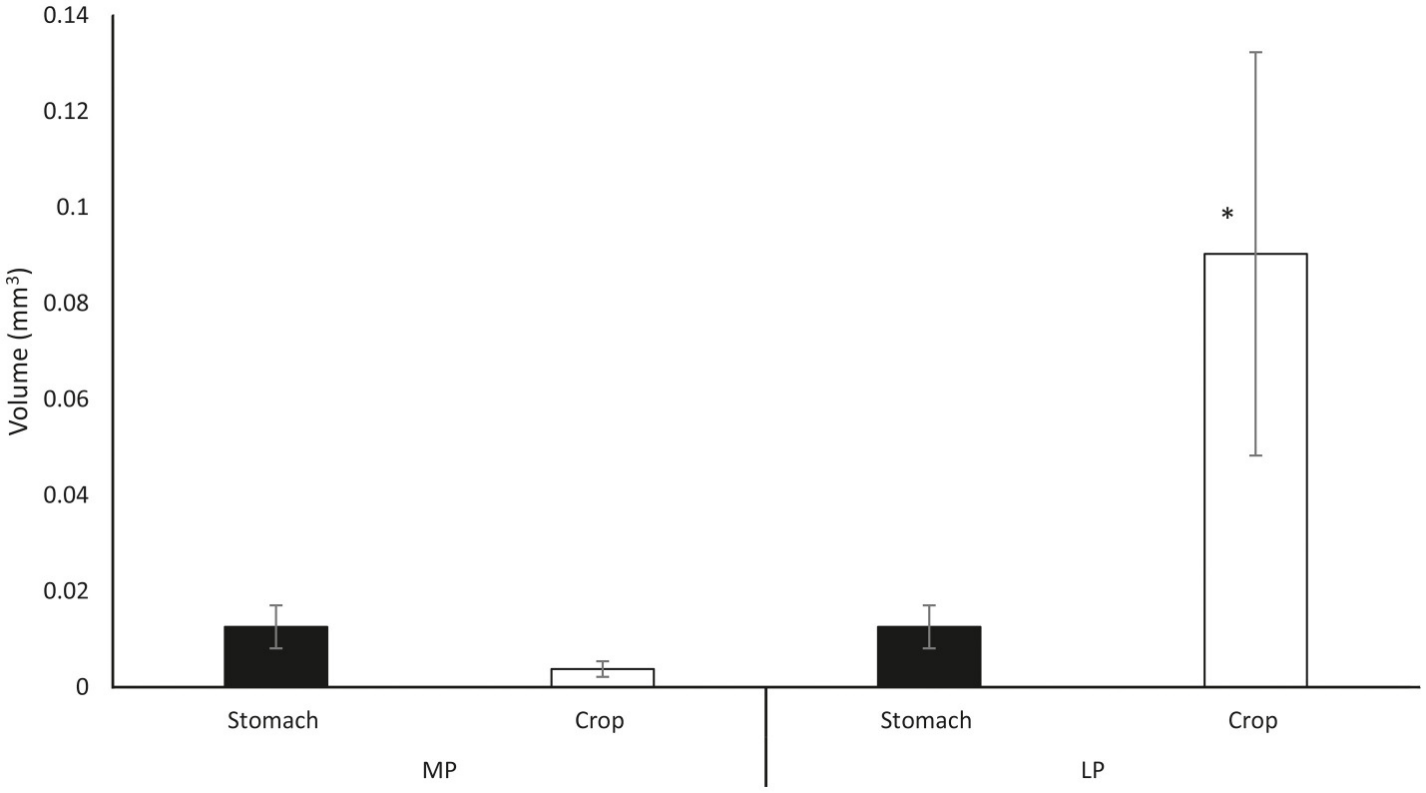
Mean crop volume (open bars) and stomach volume (filled bars) as combining data from all ingested prey types. MP measurements taken at maximum stomach volume during middle phase; LP volume measurements taken in the late ingestion phase at maximum crop volume (Note that there is no change in the gastric volume). Data plotted as mean ± *SD* (*n* = 55). The asterisk (^*^) indicates significant differences between ingestion phases (MP vs. LP) within organ (ANOVA-RM, *p* < 0.05).

Following exoskeleton penetration (after IP phase), paralarvae inserted the radula into the prey and initiated the ingestion of its internal content (Video, from second 61 to second 69, Supplementary Material). RM (radula movement) frequency in the middle phase (MP_ RM = 6.59 ± 1.7/10 s) did not differ from that in the LP (RM_LP = 6.21 ± 1.8/10 s) (ANOVA-RM, *F* = 6.65, *p* = 0.278). However, the RM frequency differed significantly in copepods and cladocerans (*n* = 45) (RM_MP_ = 4.27 ± 1.03/10 s; RM_LP_ = 4.07 ± 1.16/10 s, respectively) as compared to the rest of the species (*n* = 153) (RM_MP_ = 7.27 ± 1.11/10 s; RM_LP_ = 6.84 ± 1.05/10 s) (one-way ANOVA, *F*_MP_ = 4.72, *p* = 0.0001; *F*_LP_ = 7.02, *p* = 0.0001; Figure 3C). Food ingestion was assisted by buccal mass movements, the beak and the radula. Food passed through the esophagus into the upper digestive tract, bypassed the crop and entered the stomach where it accumulated as feeding proceeded (Video, from second 61 to second 103, Supplementary Material). Before its complete filling (defined as the volume plateauing) the stomach contracted at a frequency of 5.24 ± 0.78/10 s (Table 2 in Supplementary Material) independent of prey type (one-way ANOVA, *F* = 3.67, *p* = 0.12). Stomach contractions stopped after it had filled but the paralarvae continued feeding and accumulating food in the crop. The stomach volume at its maximum filling was Sv = 0.016 ± 0.008 mm^3^ (*n* = 56) and was prey-independent (ANOVA-RM, *F* = 16.44, *p* = 0.084; Figure 4, Table 3 in Supplementary Material).

The crop exhibited rhythmic peristaltic contractions (PC = 18 ± 6/min; *n* = 56; Video, from second 103 to second 130, Supplementary Material) to deliver food toward the stomach from where it passed into the caecum. It was not possible to define a fixed time at which food began to move from the stomach to the caecum. In some paralarvae, food transfer to the caecum began once the crop was full but in other paralarvae it began either at the end of the ingestion phase or once the paralarvae released the prey.

**Figure 5:**
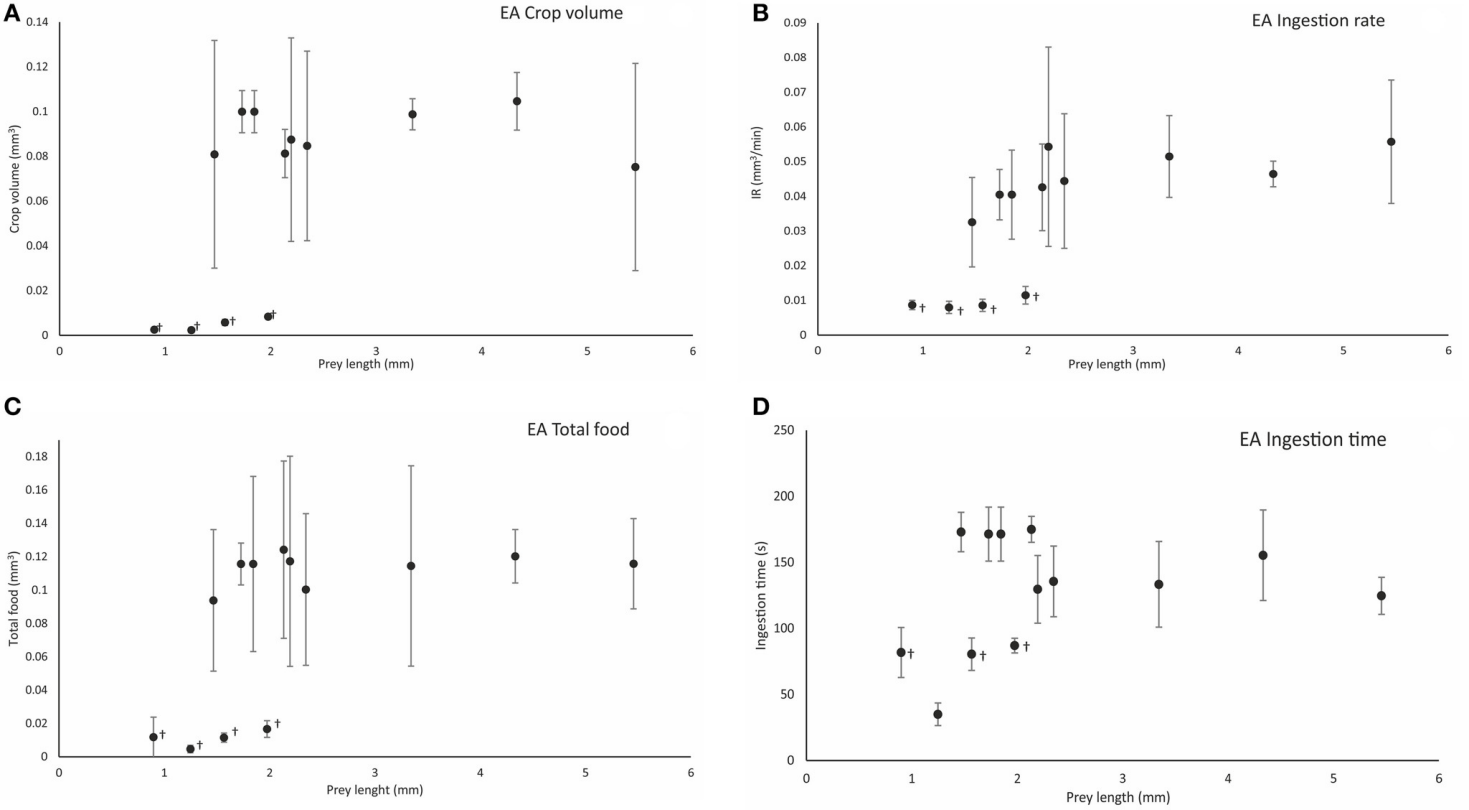
Calculated crop volume (Cv mm^3^) **(A)**, ingestion rate (IR, mm^3^/min) **(B)**, total food volume (TF, mm^3^) **(C)**, and ingestion time (IT, s) **(D)**, in relation to prey length (mm) following effective attacks (EA) on different prey types. Symbol (^†^) indicates significant differences (one-way ANOVA, *p* < 0.05) between prey types at the end of ingestion. Vertical bars represent the standard deviation of the mean of the variable (see the main text for definitions and details).

Crop volume at the end of the ingestion phase varied significantly between small prey such as copepods and cladocerans (Cv = 0.005 ± 0.003 mm^3^; *n* = 15) and large prey such as decapods and krill (Cv = 0.09 ± 0.04 mm^3^) (one-way ANOVA, *F* = 17.805, *p* = 0.0001; Figure 5A). The ingestion rate (IR) differed significantly between all copepod prey (cladocerans, *P. longicornis, C. maenas*, and *C. pagurus zoeae*; IR = 0.009 ± 0.002 mm^3^/min; *n* = 28) and the rest of prey, particularly the large ones (IR = 0.045 ± 0.007 mm^3^/min; *n* = 33; one-way ANOVA, *F* = 9.027, *p* = 0.0001; Figure 5B). Rhythmic contractile activity was observed throughout the digestive tract from the initiation of prey drilling (Video, from second 113 to second 130, Supplementary Material). Such contractions were prominent, with a high frequency (7.29 ± 0.41/10 s) in the terminal part of the intestine throughout the intake process and did not depend on prey type (one-way ANOVA, *F* = 17.805, *p* = 0.0001). Total food intake was significantly less in copepods and cladocerans (TF = 0.011 ± 0.005 mm^3^) than in larger species (TF = 0.113 ± 0.05 mm^3^) (one-way ANOVA, *F* = 41.91, *p* = 0.0001; Figure 5C). The ingestion time was significantly shorter in copepods and cladocerans (IT = 73.13 ± 23.34 s; *n* = 15) than in decapod zoeae and euphausiids (IT = 152.49 ± 29.40 s; *n* = 41) (one-way ANOVA, *F* = 15.37, *p* = 0.0001; Figure 5D). Total contact time was significantly shorter in copepods and cladocerans (TCT = 84.73 ± 21.86 s) than in the remaining prey (TCT = 220.17 ± 25.44 s) (one-way ANOVA, *F* = 31.18, *p* = 0.0001).

### Post-ingestion activity in the digestive tract

Paralarvae detached from prey in the post-ingestion phase (Video, from second 139 to second 148, Supplementary Material; ≈10 min recording time). Mantle contraction frequency decreased significantly between the ingestion phase (MC = 13.37 ± 0.13/10 s; *n* = 56) and the post ingestion phase (MC = 6.07 ± 0.67/10 s) (ANOVA-RM, *F* = 989.33, *p* = 0.00001). During this latter phase, the crop was fully distended and showed intermittent peristaltic activity characterized by episodes of contractile activity lasting 2.0 ± 1.0 s at a frequency 3.0 ± 1.0 /10 s and at intervals of 6.58 ± 1.85 s (*n* = 34) between episodes (Video, from second 150 to second 176, Supplementary Material). The caecum contained prey pigments (reddish from *T. longicornis* and blackish for *C. maenas*) which colored the digestive gland as food entered the hepatopancreatic duct. Food began entering the digestive gland *via* the hepatopancreatic duct 312 ± 32 s after the crop was full (Video, from second 176 to second 210, Supplementary Material). Contents from the digestive tract not entering the digestive gland proceeded along the intestine and were expelled through rectum and anus. During the latter activity, the terminal part of the intestine contracted at frequency of 8.87 ± 1.46/10 s which did not differ from that observed during prey ingestion (ANOVA-RM, *F* = 37.34, *p* = 0.001). Feces were gray in color and string-like in appearance (Video, from second 210 to second 250, Supplementary Material). During defaecation (observed in 12% of specimens) the MC frequency increased to 14.23 ± 0.44/10 s (*n* = 4), a value comparable to that observed during the ingestion phase (13.37 ± 0.13/10 s; Video, from second 210 to second 250, Supplementary Material).

### Digestive tract activity during ineffective attacks

**Figure 6:**
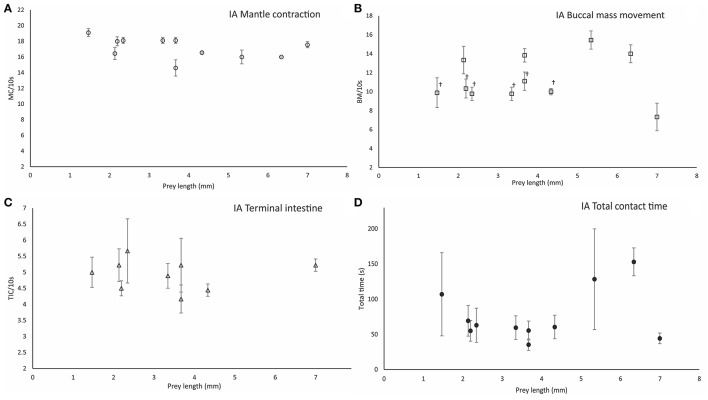
Parameters measured during ineffective attacks (IA). Mantle contraction frequency (MC/10 s) **(A)**, buccal mass movement frequency (BM/10 s) **(B)**, terminal intestine contraction frequency (TIC/10 s) **(C)**, and total contact time between paralarvae and prey (TCT/10s) **(D)**. Prey types are grouped by prey size in length (mm). Symbols (^†^) indicate significant differences (one-way ANOVA, *p* < 0.05) between prey types. Vertical bars represent the standard deviation of the mean for each variable.

The frequency of mantle contractions during ineffective attacks did not differ between PPC phases (MC_IP_ = 17.37 ± 1.44/10 s; MC_MP_ = 16.86 ± 1.80; and MC_LP_ = 17.4 ± 1.83/10 s) (ANOVA-RM, *F* = 3.44, *p* = 0.33), and averaged 17.75 ± 1.01/10 s (*n* = 105) overall (Figure 6A). The frequency of MC did not differ among prey types (one-way ANOVA, *F* = 1.177, *p* = 0.319) or between the initial phase of effective attacks (MC_IP−EA_ = 17.08 ± 1.44/10 s, *n* = 66; see above) vs. ineffective attacks (MC_IP−IA_ = 17.75 ± 1.01/10 s) (one-way ANOVA, *F* = 0.28, *p* = 0.60). However, MC frequency was significantly lower during the middle-phase of ineffective attacks as compared to effective attacks (MC_MP−IA_ = 13.67 ± 1.43/10 s; *n* = 66; one-way ANOVA, *F* = 198.05, *p* = 0.0001).

Following attack and prey holding (i.e., once PPC was established), paralarvae grasped the prey accompanied by lateral shaking of head and arms and siphon jetting propulsions (SP = 3.0 ± 1.0/10 s; *n* = 105), and attempted to pierce the exoskeleton with the beak. The buccal mass movements were at a frequency MC = 11.14 ± 2.37/10 s, MC = 10.89 ± 2.85/10 s, and MC = 11.11 ± 2.75/10 s in phases IP, MP, and LP respectively, (Table 4 in Supplementary Material). BM frequency did not differ among ingestion phases (one-way ANOVA, *F* = 0.098, *p* = 0.910) and averaged BM = 11.05 ± 2.64/10 s (*n* = 105). BM frequency differed between Crangonidae, megalopae and Gammaridae amphipods (BM = 13 ± 1.41 /10 s) and *P. longicornis* (BM = 9.89 ± 1.58/10 s) (one-way ANOVA, *F* = 16.903, *p* = 0.00001; Figure 6B). BM frequency differed significantly between EA (BM = 11.14 ± 1.37/10 s) and IA (BM = 9.075 ± 1.57/10 s) in the initial phase of ingestion (one-way ANOVA, *F* = 5.82, *p* = 0.0001). Although, paralarvae were unable to pierce the prey in ineffective attacks, rhythmic contractile activity was observed in their intestine. The frequency of rhythmic contractions did not differ between prey types (one-way ANOVA, *F*_IP_ = 0.96, *p* = 0.49; *F*_MP_ = 1.91, *p* = 0.11; *F*_LP_ = 1.6, *p* = 0.18) at any phase (ANOVA-RM, *F* = 4.33, *p* = 0.07) and averaged 5.24 ± 1.23/10 s (*n* = 87; Figure 6C). Significant differences were observed between the higher terminal intestine contraction frequency recorded during EA (7.34 ± 0.92/10 s) and that observed during IA (5.24 ± 1.23/10 s; one-way ANOVA, *F* = 342.03, *p* = 0.0001). Total contact time between paralarvae and prey (TCT) varied significantly with prey type (one-way ANOVA, *F* = 3.73, *p* = 0.004; *n* = 35). The shortest TCT (35.33 ± 8.14 s) was observed in gastropods and the longest TCT (153 ± 19.78 s) was observed in gammarids (Figure 6D). TCT was significantly higher in EA attacks as compared to IA attacks (one-way ANOVA, *F* = 81.84, *p* = 0.00001). For instance, TCT = 230.6 ± 17.89 s during EA on *M. brachydactyla* zoeae and TCT = 69.34 ± 21.78 s when an ineffective attack occurred on the same prey (Video, from second 250 to second 303, Supplementary Material).

## Discussion

The general aim of this study was to understand the feeding mechanisms employed by paralarvae of the common octopus fed different prey types and the physiological dynamics operating during digestion. Specific results for each of the objectives outlined in the Introduction are discussed in relation to knowledge of digestive tract physiology from adult cephalopods.

### Paralarvae attack and prey defensive strategies

The first specific objective of this study was the *in vivo* quantification of the attack strategy and the related behavior exhibited by octopus paralarvae on wild zooplankton and zoeae of spider crab and edible crab hatched from the broodstock. *O. vulgaris* paralarvae coexist in the wild with diverse zooplankton, i.e., copepods, cladocerans, zoeae of different decapods, mysidacea, euphausiids, gammarids and hyperiids amphipods, gastropods, cirripedia nauplii (Roura et al., 2013), the smallest among them captured and ingested by paralarvae, i.e., cladocerans (*P. intermedius*, length 0.90 ± 0.01 mm) and copepods (*A. clausii* and *T. longicornis*, length 1.25 ± 0.11 and 1.57 ± 0.12 mm, respectively). The ability of octopus paralarvae to capture copepods (Nande, 2016) and cladocerans is confirmed herein. Nevertheless, occasional failed attacks on copepods was due to their escape reaction upon detection the predator movements (Yen et al., 1992; Fields and Yen, 1997; Paffenhöfer, 1998) as has also been observed in paralarvae of the squid, *Loligo opalescens* (Chen et al., 1996).

Notably, current results also indicate that 3 dph paralarvae are able to effectively feed on both, small and large live prey and not only on prey of length >2 mm (Iglesias et al., 2006). The highest percentage of EA among decapods zoeae took place on zoeae of *C. maenas, M. brachydactyla*, and *C. pagurus* (80, 86, and 83%, respectively). Consistent with those data, the best growth and survival rates reported in recent years from rearing experiments of *O. vulgaris* paralarvae were obtained using zoeae of *M. brachydactyla* (Iglesias et al., 2004; Carrasco et al., 2006), *Grapsus grapsus* and *Plagusia depressa* (Iglesias et al., 2007), *Liocarcinus depurator* and *Pagurus prideaux* (Villanueva, 1994, 1995). Also, diverse analyses of stomach content from wild paralarvae contained species of decapods zoeae such as *Necora puber, Polybius henslowii, Pirimela denticulata*, among others (Roura et al., 2012). Other prey types such as zoeae of families Paguridae, Processidae, Hippolytidae, and Palaemonidae were also captured by octopus paralarvae with a lower efficiency of attacks (60–75%) as compared to zoeae of the preferred species.

The cephalothorax area was the site targeted in all effective attacks (irrespective of prey type) but ineffective attacks occurred on the abdomen (e.g., on krill species). Supportive evidence for the hypothesis of a deliberate targeting of the prey cephalothorax is the number of effective attacks on that body area. A less plausible but untested hypothesis comes from nutritional analyses of krill (*Meganyctiphanes norvegica*) and states that the cephalothorax area contains a higher proportion of food per volume and a different lipid profile as compared to the abdomen (Albessard et al., 2001). Although, paralarvae directed their attacks toward the cephalothorax, about 25% of them were ineffective because of the escape ability of the prey after their initial abdominal contact. For instance, zoeae of *C. maenas, C. pagurus*, and *M. brachydactyla* exposed to paralarvae exhibited a defensive behavior consisting on rapid swimming movements and fast spinning, in agreement with previous observations (Hernández-García et al., 2000). Two alternatives to escaping consisted of facing the predator using sharp spines which occasionally caused mortal injuries to the paralarvae (Villanueva and Norman, 2008) and the use of chelipeds as defense tools (*Brachiura megalopa*) as observed in current and previous studies (Bertini and Fransozo, 1999).

A 100% frequency of ineffective attacks was observed in all species of families Crangonidae, gastropods, Gammaridae and Hyperiidae amphipods and Brachiura megalopae, irrespective of the body area targeted. Those IA were due to the inability of the paralarvae to pierce the exoskeleton of those prey (see Figure 2). Although, some Cragnonidae prey were previously reported from stomach contents of wild paralarvae (Roura et al., 2012), current observations show that a 3 dph beak is relatively smaller than that of older paralarvae (Perales-Raya et al., 2014) and therefore it is practically ineffective on mineralized exoskeletons.

Mantle contractions are related to swimming and breathing (Villanueva, 1994; Villanueva and Norman, 2008). During the initial phase of PPC, the mantle contraction frequency was ~100/min and progressively decreased during ingestion and post-ingestion to ~75/min. Studies of *O. vulgaris* (at 15°C) ranging in weight from 2.5 g to 8 Kg showed a progressive reduction in respiration rate from 51/min to 12/min (Polimanti, 1913, cited in Wells, 1978, Table 3.1, p26). That range is consistent with baseline values for larger octopuses in the later literature (~150 g to ~2 Kg, ~16 bpm to ~30 b/min; Andrews and Tansey, 1981; Wells and Wells, 1985; Valverde and García, 2005). The higher mantle contraction frequency of paralarvae as compared to specimens weighing >2.5 g is not odd since the metabolic rate is expected to be higher in rapidly developing paralarvae as compared to adults (Iglesias et al., 2004; Semmens et al., 2004). Current data suggest that the lower frequency of mantle contractions relates to a precise prey manipulation, as observed in older octopus paralarvae (Villanueva et al., 1996). Indeed, paralarvae successfully positioned themselves as well as the prey using combined contractions of mantle and siphon, in addition to movements of head and arms. Those actions need coordination between the central nervous system and the visual system (see Figure 2) and the mechanisms by which this is achieved require further research.

### Prey drilling and ingestion dynamics

The second specific objective of this study was the *in vivo* quantification of the dynamics of both, exoskeleton penetration (“drilling”) and ingestion of different prey types. Monitoring those processes was feasible thanks to the transparency of paralarvae (Nande et al., 2017) which allowed the use of high resolution video-recording to characterize and quantify the mechanisms for the first time in a live cephalopod. Current results show that during the initial phase, when paralarvae attempted to drill the prey, it used buccal mass movements (Villanueva and Norman, 2008) which were prey-dependent. The BM frequency was significantly lower when feeding on copepods and cladocerans bearing a less chitinized exoskeleton than on prey with a thicker, more mineralized exoskeleton (e.g., 6.16 ± 2.64/10 s vs. 9.24 ± 1.29/10 s). Consequently, BM frequency was higher (11.05 ± 2.64/10 s) during the initial phase of ineffective attacks on mineralized exoskeletons (amphipods, Crangonidae, Brachiura megalopae, and Gammaridae).

In effective attacks, radula movements (RM) began after prey drilling and proceeded at constant frequency (6.59 ± 1.7/10 s). However, RM frequency was higher on large prey than on small prey, particularly in the later phases. We were unable to disentangle the relative roles that the beak, the radula and the buccal mass played during ingestion, but it is assumed that food particles moved into the esophagus by the coordinated activity of the above three structures. In this regard, Altman and Nixon (1970) reported that ingestion of crab in adult octopus was possible in radula-less specimens by using the lateral buccal palps. Nevertheless, those authors also reported that radula-less specimens were unable to perform the “*more delicate parts of the cleaning process*” (Altman and Nixon, 1970, p. 35). Therefore, it is likely that the radula is important during ingestion requiring the coordination of beak, radula, and buccal mass muscles by the inferior buccal and subradular ganglia (Boyle et al., 1979a,b). Lesion studies have shown that eating also requires connection of those ganglia with the superior buccal ganglia (Young, 1965) and current observations suggest that the inferior buccal, subradular and superior buccal ganglia are sufficiently mature in 3 dph octopus paralarvae to coordinate food ingestion. However, the possibility exists that in such an immature state, ingestion may be regulated solely by the peripheral ganglia as triggered by buccal contact with prey.

Food passed from the buccal cavity into the relatively narrow esophagus where peristaltic contractions conveyed it to crop and stomach (Andrews and Tansey, 1981). The total contact time between prey and paralarvae was short (<5 min) so ingestion was relatively rapid. Therefore, the rapid food ingestion and storage of paralarvae is particularly adaptive because of the high vulnerability of feeding paralarvae to predation. Since attack effectiveness and subsequent ingestion were prey-dependent, paralarvae exhibited an optimized strategy for prey capture, drilling, and ingestion time upon potential food energy intake, density and digestibility. Current data provides a basis for considering how octopus paralarvae feeding fits with published models of feeding (Schoener, 1971), particularly for invertebrate larvae (e.g., Crustacea, Le Vay et al., 2001).

### Post ingestion digestive tract motility

The third specific objective of this study was the *in vivo* quantification of the distribution of food into the crop, the stomach, and the digestive gland as well as the characterization of the motility patterns. The crop is an elongated sack-like structure located between the esophagus and the stomach of Octopoda (e.g., *O. vulgaris*, Andrews and Tansey, 1983; *O. maya*, Linares et al., 2015; *Octopus cyanea*, Boucher-Rodoni, 1973) and Nautiloids (e.g., *Nautilus pompilius*, Owen, 1832; Westermann and Schipp, 1998; Westermann et al., 2002) which is absent from Sepidae and Teuthoidea where the ingested food passes directly to the stomach (for reviews see: Bidder, 1966; Boucaud-Camou and Boucher-Rodoni, 1983). Studies on adult *O. vulgaris* (Andrews and Tansey, 1983) and *N. pompilius* (Westermann et al., 2002) provided evidence that the crop is adapted for food storage. Such storage function in *O. vulgaris* is facilitated by the thin muscular wall of the crop and by suppression of the contractile activity proposed to be cholinergic (Andrews and Tansey, 1983). *Post mortem* analysis of *O. vulgaris* led to the suggestion that the initial food storage (accommodation) during feeding occurred in the crop (Bidder, 1957; Young, 1960; Wells, 1978; Andrews and Tansey, 1983), then gradually moving toward the stomach where it would be triturated by the reciprocal movement of two thick apposed muscular blocks (Andrews and Tansey, 1983). Current observations on crop and stomach filling in paralarvae challenges the above classical view of the crop-stomach relationship. In the early ingestion phase of paralarvae (MP) the stomach had a higher volume than the crop (Figure 4) and its volume plateaued as ingestion proceeded (MP) while the crop volume continued to increase. At the end of feeding (LP, late phase) the crop volume was ~10x its volume as measured at the end of the MP (when the stomach volume had already plateaued). These results show that although the crop is the location where the majority of ingested food is stored in paralarvae, the first food ingested proceeds straight to the stomach. Interestingly, by the time the stomach volume plateaued (MP, Figure 4) and the crop began to fill (phase transition MP to LP), the contractions of the stomach ceased, suggesting a coordinated activity between the crop and the stomach. The gastric ganglion is most likely responsible for such coordination as stimulation of the ganglion in adult octopus can simultaneously inhibit gastric contractions and enhance crop activity (Andrews and Tansey, 1983). Also, *in vitro* studies provide evidence that the contractile activity of crop and stomach of cephalopods is mediated by catecholamines (Bacq, 1934; Wood, 1969; Andrews and Tansey, 1983).

Since the time between prey contact and the end of ingestion did not differ within prey among individuals, a putative satiety signal could exist. One possibility is a sensitive buccal signal upon the full removal of prey content. However, a more plausible explanation is the distension of the crop as the triggering signal for feeding termination as suggested by Nixon (1966) in adult octopus. While digestive tract mechano- and chemo- receptive afferents signaling from the digestive tract to the brain are well established in vertebrates (e.g., Andrews, 1986; Olsson, 2011; Brookes et al., 2013), evidence of afferents from the crop and other regions of the digestive tract projecting to the brain is very limited in cephalopods (Young, 1965, 1971). The possibility that food ingestion could release gut hormones to act on the brain to terminate feeding, as occurs in vertebrates (e.g., Dockray, 2014; Volkoff, 2016) should not be overlooked.

There is no evidence of a sphincter placed between the crop and the stomach in adult *O. vulgaris*, so ingested food (fish or crab) can move in either direction depending on the digestive tract contractile activity (Andrews and Tansey, 1983). It has been proposed that crop and stomach should be regarded as a functional unit coordinated by the gastric ganglion. In such a model, the crop accommodates food and delivers it to the stomach where it is triturated by the muscle blocks and mixed with digestive secretions. Food can then be returned to the crop or delivered to the distal digestive tract, depending on its degree of digestion (Andrews and Tansey, 1983). The nature of the ingested food in paralarvae as compared to adult octopus could explain why such repeated cycling between crop and stomach proposed in adults may not apply in paralarvae, i.e., the higher food digestibility and the low amount (if any) of indigestible residuals.

The peristaltic contractions of the crop moved food to the stomach with periods of contractile activity interspersed with quiescence. The frequency of crop contractions was ~18/min in paralarvae what is similar to the range of 10–20/min for small amplitude contractions recorded *in vitro* from longitudinal muscle in adult *O. vulgaris* (Andrews and Tansey, 1981). That *in vitro* study also recorded sustained (10–20 s) large amplitude contractions which were not obvious in the present study. Such lack of correspondence between studies regarding duration of contractions can be due to the shorter recording time of the current study. However, the faster passage of food throughout the digestive tract of paralarvae as compared to adults cannot be overlooked. Further recording of crop activity using food marked with fluorescent microspheres might facilitate understanding the relationship between the external appearance of the crop and the movement of its content. The stimulus for initiation of crop contractions is not known but *in vitro* studies show that distension would stimulate contractions in the crop (Andrews and Tansey, 1983). However, there must be coordination between the crop and the stomach which most likely operates via the innervation of the various gut regions from the gastric ganglion (Young, 1967), which is able to modulate both, stomach and crop motility (Andrews and Tansey, 1983).

The contraction frequency of the full stomach was ~30/min when full, i.e., when food began moving from the stomach to the caecum. Such a frequency is approximately twice the one recorded *in vitro* in adult octopus (Andrews and Tansey, 1983). Subsequently, pigmented food material was observed to move from the caecum to the digestive gland via the hepatopancreatic duct. Although, we were unable to track specific food particles during transit from the crop to the stomach, pigmented particles were seen to enter the digestive gland ~5 min after the crop filled. Therefore, the total time between food ingestion until it enters the digestive gland in paralarvae is in the range of minutes as opposed to hours in adult octopus (Bidder, 1957, 1966; Boucaud-Camou et al., 1976; Linares et al., 2015).

The terminal part of the intestine showed rhythmic contractile activity during all phases (even during ineffective attacks). An intestine contraction frequency of ~50/min is high for a tissue assumed to be composed of smooth muscle (no data available in paralarvae) and is well above the range of ~12 to ~18/min recorded *in vitro* in the rectum and intestine of adults (Andrews and Tansey, 1983). Defaecation was a rare event, since it was only recorded in 4 animals out of 34 (~12%) but the string-like appearance of feces was similar to that reported in adult *O. vulgaris* (Bidder, 1957). Defaecation in adult *O. vulgaris* is accompanied by changes in respiration (Wells, 1978) and was accompanied by an increase in the frequency of mantle contractions in paralarvae. Such activity suggests the involvement of central nervous system coordination between the distal digestive tract (regulated by the atriorectal nerve from the palliovisceral brain lobe (Young, 1967, 1971) and the somatomotor system of the mantle muscle (Wells, 1978).

## Conclusions

This paper originally provides a detailed quantification of the attack and ingestion of a range of live prey by *O. vulgaris* paralarvae at a very early post-hatching stage. The first two goals show that effective attacks targeted vulnerable regions of the prey and that dynamics of buccal mass, radula movements, ingestion time and mantle contraction, suggest that paralarvae receive feedback from the prey exoskeleton and its inner content. The third goal indicates that the process of ingestion and transfer to the digestive gland for assimilation is faster than in adults. These results establish the utility of high-resolution video recording of paralarvae as a real-time method for studying the motility of the digestive tract (although limited by relatively short recording times and paralarvae orientation) and suggest that the dogma about crop-stomach relationships in adults may need reconsideration. As paralarvae remain transparent until settlement, this method should enable tracking the full maturation of digestive tract function.

This study provides a new perspective on feeding strategies that could be adopted in octopus aquaculture, where octopus paralarvae survival remains an issue. Therefore, three dph paralarvae would need to consume tenfold more copepods as compared to zoeae in order to obtain a food equivalent. It is critical that paralarvae are fed the adequate live prey, which they can drill and ingest better than *a priori* appealing Crangonidae or Gammaridae species equipped with inaccessible exoskeletons. The characterization of digestive tract function described here (e.g., ingestion rate, motility, time for food entry into the digestive gland, and defaecation) permits the assessment of the digestibility of different prey types to improve paralarvae growth and survival.

## Author contributions

MN, MP, and PP worked out the conception, experimental design and execution of this study. All the authors (MN, PP, AR, PA, and MP) contributed significantly to achieve this publication, e.g., discussed the results and implications, commented on the manuscript at all stages and finally approved its submission for publication in Frontiers of Physiology in the Research Topic “The Digestive Tract of Cephalopods: at the Interface Between Physiology and Ecology.”

### Conflict of interest statement

The authors declare that the research was conducted in the absence of any commercial or financial relationships that could be construed as a potential conflict of interest.

## References

[B1] Albessard E. Mayzaud P. Cuzin-Roudy J. (2001). Variation of lipid classes among organs of the northern krill *Meganyctiphanes norvegica*, with respect to reproduction. Comp. Biochem. Physiol. Part A Mol. Integr. Physiol. 129, 373–390. 10.1016/S1095-6433(00)00355-X 11423310

[B2] Altman J. S. Nixon M. (1970). Use of beaks and radula by *Octopus vulgaris* in feeding. J. Zool. 161, 25–38. 10.1111/j.1469-7998.1970.tb02167.x

[B3] Andrews P. L. R. (1986). Vagal afferent innervation of the gastrointestinal tract. Prog. Brain Res. 67, 65–86. 10.1016/S0079-6123(08)62757-0 3823483

[B4] Andrews P. L. R. Darmaillacq A. S. Dennison N. Gleadall I. G. Hawkins P. Messenger J. B. (2013). The identification and management of pain, suffering and distress in cephalopods, including anaesthesia, analgesia and humane killing. J. Exp. Mar. Bio. Ecol. 447, 46–64. 10.1016/j.jembe.2013.02.010

[B5] Andrews P. L. R. Tansey E. M. (1981). The effects of some anesthetic agents in *Octopus vulgaris*. Comp. Biochem. Physiol. C 70, 241–247. 10.1016/0306-4492(81)90057-5

[B6] Andrews P. L. R. Tansey E. M. (1983). The gastrointestinal tract of *Octopus vulgaris*: a re-examination of the anatomy physiology and pharmacology of the upper tract. J. Mar. Biol. Assoc. 63, 109–134. 10.1017/S0025315400049845

[B7] Bacq Z. M. (1934). Recherches sur la physiologie du systéme nerveux autonome V. Reactions du ventricle median, des chromatophores et de divers organes isoles d'un mollusque cephalopode (*Loligo pealeii*) A l'adrenaline, l'acetylcholine, l'ergotamine, l'atropine et aux ions K, Ca et Mg. Archiv. Int. Physiol. XXXVIII, 138–159.

[B8] Baeza-Rojano E. Domingues P. Guerra-García J. M. Capella S. Noreña-Barroso E. Caamal-Monsreal C. (2013). Marine gammarids (Crustacea: Amphipoda): a new live prey to culture *Octopus maya* hatchlings. Aquacult. Res. 44, 1602–1612. 10.1111/j.1365-2109.2012.03169.x

[B9] Bertini G. Fransozo A. (1999). Relative growth of *Petrochirus diogenes* (Linnaeus, 1758) (Crustacea, Anomura, Diogenidae) in the Ubatuba region, São Paulo, Brazil. Rev. Bras. Biol. 59, 617–625. 10.1590/S0034-71081999000400011 23505650

[B10] Bidder A. M. (1957). Evidence for an absorptive function in the liver of *Octopus vulgaris*. Pubbl. Staz. Zool. Napoli 29, 139–150.

[B11] Bidder A. M. (1966). Feeding and digestion in cephalopods, in Physiology of the Mollusca, ed Yonge C. M. (New York, NY: Academic Press Inc.), 97–124. 10.1016/B978-1-4832-3242-3.50009-4

[B12] Boucaud-Camou E. Boucher-Rodoni R. (1983). Feeding and digestion in cephalopods, in The Mollusca - Physiology, Part 2, eds Saleuddin A. S. M. Wilbur K. M. (New York, NY: Academic Press), 149–187. 10.1016/B978-0-12-751405-5.50011-7

[B13] Boucaud-Camou E. Boucher-Rodoni R. Mangold K. (1976). Digestive absorption in *Octopus vulgaris* (Cephalopoda: Octopoda). J. Zool. 179, 261–271. 10.1111/j.1469-7998.1976.tb02295.x

[B14] Boucher-Rodoni R. (1973). Vitesse de digestion d'*Octopus cyanea* (Cephalopoda: Octopoda). Mar. Biol. 18, 237–242. 10.1007/BF00367990

[B15] Boyle P. R. Mangold K. Froesch D. (1979a). The mandibular movements of *Octopus vulgaris*. J. Zool. 188, 53–67. 10.1111/j.1469-7998.1979.tb03392.x

[B16] Boyle P. R. Mangold K. Froesch D. (1979b). The organization of beak movements in *Octopus*. Malacologia 18, 423–430.

[B17] Brookes S. J. Spencer N. J. Costa M. Zagorodnyuk V. P. (2013). Extrinsic primary afferent signalling in the gut. Nat. Rev. Gastroenterol. Hepatol. 10, 286–296. 10.1038/nrgastro.2013.29 23438947

[B18] Carrasco J. F. Arronte J. C. Rodríguez C. (2006). Paralarval rearing of the common octopus, *Octopus vulgaris* (Cuvier). Aquac. Res. 37, 1601–1605. 10.1111/j.1365-2109.2006.01594.x

[B19] Chen D. S. Dykhuizen G. V. Hodge J. Gilly W. F. (1996). Ontogeny of copepod predation in juvenile squid (*Loligo opalescens*). Biol. Bull. 190, 69–81. 10.2307/1542676 8852631

[B20] Dockray G. J. (2014). Gastrointestinal hormones and the dialogue between gut and brain. J. Physiol. 592, 2927–2941. 10.1113/jphysiol.2014.270850 24566540 PMC4214649

[B21] Doubleday Z. A. Prowse T. A. Arkhipkin A. Pierce G. J. Semmens J. Steer M.. (2016). Global proliferation of cephalopods. Curr. Biol. 26, R406–R407. 10.1016/j.cub.2016.04.002 27218844

[B22] Estévez A. Gairin I. Berger E. (2009). Wild zooplancton for *Octopus vulgaris* larval rearing, in LARVI 09, Fish & Shellfish Larviculture Symposium, Special Publication No. 38. eds Hendry C. I. Van Stappen G. Wille M. Sorgeloos P. (Oostende: European Aquaculture Society), 88–91.

[B23] European Parliament Council of the European Union (2010). Directive 2010/63/EU of the European Parliament and of the Council of 22 September 2010 on the Protection of Animals Used for Scientific Purposes. Strasbourg: Council of Europe.

[B24] Fields D. M. Yen J. (1997). The escape behavior of marine copepods in response to a quantifiable fluid mechanical disturbance. J. Plankton Res. 19, 1289–1304. 10.1093/plankt/19.9.1289

[B25] Fiorito G. Affuso A. Anderson D. B. Basil J. Bonnaud L. Botta G.. (2014). Cephalopods in neuroscience: regulations, research and the 3Rs. Invert. Neurosci. 14, 13–36. 10.1007/s10158-013-0165-x 24385049 PMC3938841

[B26] Fiorito G. Affuso A. Basil J. Cole A. de Girolamo P. D'Angelo Ludovic D.. (2015). Guidelines for the care and welfare of cephalopods in research–a consensus based on an initiative by CephRes, FELASA and the Boyd Group. Lab. Anim. 49(2 Suppl.), 1–90. 10.1177/0023677215580006 26354955

[B27] Fiorito G. Gherardi F. (1999). Prey-handling behaviour of *Octopus vulgaris* (Mollusca, Cephalopoda) on bivalve preys. Behav. Res. 46, 75–88. 10.1016/S0376-6357(99)00020-0 24925500

[B28] Fiorito G. Scotto P. (1992). Observational learning in *Octopus vulgaris*. Science 256:545. 10.1126/science.256.5056.545 17787951

[B29] Forsythe J. W. Hanlon R. T. (1980). A closed marine culture system for rearing *Octopus joubini* and other large-egged benthic octopods. Lab. Anim. 14, 137–142. 10.1258/002367780780942737 7431823

[B30] Guerra A. Nixon M. (1987). Crab and mollusc shell drilling by *Octopus vulgaris* (Mollusca: Cephalopoda) in the Ria de Vigo (north-west Spain). J. Zool. 211, 515–523. 10.1111/j.1469-7998.1987.tb01549.x

[B31] Hanlon R. T. Messenger J. B. (1996). Cephalopod Behaviour. Cambridge: Cambridge University Press.

[B32] Hernández-García V. Martín A. Y. Castro J. J. (2000). Evidence of external digestion of crustaceans in *Octopus vulgaris* paralarvae. J. Mar. Biol. Assoc. U.K. 80, 559–560. 10.1017/S0025315400002320

[B33] Hochner B. (2012). An embodied view of octopus neurobiology. Curr. Biol. 22, R887–R892. 10.1016/j.cub.2012.09.001 23098601

[B34] Hochner B. Shomrat T. Fiorito G. (2006). The octopus: a model for a comparative analysis of the evolution of learning and memory mechanisms. Biol. Bull. 210, 308–317. 10.2307/4134567 16801504

[B35] Iglesias J. Fuentes L. (2014). Chapter 23: *Octopus vulgaris*. Paralarvae culture, in Cephalopod Culture, eds Iglesias J. Fuentes L. Villanueva R. (New York, NY; Heidelberg; Dordrecht; London: Springer), 427–450.

[B36] Iglesias J. Fuentes L. Sánchez J. Otero J. J. Moxica C. Lago M. J. (2006). First feeding of *Octopus vulgaris* Cuvier, 1797 paralarvae using Artemia: effect of prey size, prey density and feeding frequency. Aquaculture 261, 817–822. 10.1016/j.aquaculture.2006.08.002

[B37] Iglesias J. Otero J. J. Moxica C. Fuentes L. Sánchez F. J. (2004). The completed life cycle of the octopus (*Octopus vulgaris*, Cuvier) under culture conditions: paralarvae rearing using Artemia and zoeae, and first data on juvenile growth up to eight months of age. Aquacult. Int. 12, 481–487. 10.1023/B:AQUI.0000042142.88449.bc

[B38] Iglesias P. Picón P. Nande M. Lago M. J. Otero J. J. Trujillo V. (2016). Effect of low salinity on survival and ingested food of the common octopus, *Octopus vulgaris Cuvier*, 1797. J. Appl. Aquacult. 28, 267–271. 10.1080/10454438.2016.1190953

[B39] Iglesias J. Sánchez F. J. Bersano J. F. G. Carrasco J. F. Dhont J. Fuentes L. (2007). Rearing of *Octopus vulgaris* paralarvae: present status, bottlenecks and trends. Aquaculture 266, 1–15. 10.1016/j.aquaculture.2007.02.019

[B40] Le Vay L. Jones D. A. Puello-Cruz A. C. Sangha R. S. Ngamphongsai C. (2001). Digestion in relation to feeding strategies exhibited by crustacean larvae. Comp. Biochem. Physiol. Part A Mol. Integr. Physiol. 128, 621–628. 10.1016/S1095-6433(00)00339-1 11246049

[B41] Linares M. Caamal-Monsreal C. Olivares A. Sanchez A. Rodriguez S. Zuniga O. (2015). Timing of digestion, absorption and assimilation in octopus species from tropical (*Octopus maya*) and subtropical-temperate (*O. mimus*) ecosystems. Aquat. Biol. 24, 127–140. 10.3354/ab00642

[B42] Lotze H. K. Coll M. Dunne J. A. (2011). Historical changes in marine resources, food-web structure and ecosystem functioning in the Adriatic Sea, Mediterranean. Ecosystems 14, 198–222. 10.1007/s10021-010-9404-8

[B43] Nande M. (2016). Aspectos Metabólicos Clave de Nutrición Endógena y Diversificación nutricional Sobre el Desarrollo Temprano de Nuevas Especies de Acuicultura. Doctoral thesis, Universidad of Vigo, Spain Available online at: https://www.educacion.es/teseo/mostrarRef.do?ref$=$1266843

[B44] Nande M. Iglesias J. Domingues P. Pérez M. (2017). Effect of temperature on energetic demands during the last stages of embryonic development and early life of *Octopus vulgaris* (Cuvier, 1797) paralarvae. Aquac. Res. 48, 1951–1961. 10.1111/are.13032

[B45] Navarro J. C. Monroig O. Sykes A. V. (2014). Nutrition as a key factor for cephalopod aquaculture, in Cephalopod Culture, eds Iglesias J. Fuentes L. Villanueva R. (New York, NY: Springer), 77–96.

[B46] Nixon M. (1966). Changes in body weigt and intake of food by *Octopus vulgaris*. J. Zool. 158, 475–483. 10.1111/j.1469-7998.1969.tb02163.x

[B47] Nixon M. (1968). Feeding Mechanisms and Growth in 'Octopus vulgaris'. Doctoral dissertation, Ph.D. Degree, University of London.

[B48] Norman M. D. Finn J. K. Hochberg F. G. (2014). Family octopodidae, in Cephalopods of the World. An Annotated and Illustrated Catalogue of Cephalopod Species Known to Date. Octopods and Vampire Squids. FAO Species Catalogue for Fishery Purposes. No. 4., eds Jereb P. Roper C. F. E. Norman M. D. Finn J. K. (Rome: Food and Agriculture Organization of the United Nations), 36–215.

[B49] O'dor R. K. Mangold K. Boucher-Rodoni R. Wells M. J. Wells J. (1984). Nutrient absorption, storage and remobilization in *Octopus vulgaris*. Mar. Freshw. Behav. Physiol. 11, 239–258. 10.1080/10236248409387049

[B50] Olsson C. (2011). The enteric nervous system, in Fish Physiology, eds Martin Grosell A. P. F. Colin J. B. (London, UK: Academic Press), 319–349.

[B51] Owen R. (1832). Memoir on the Pearly Nautilus: (Nautilis, Pompilius, Linn.), with Illustrations of Its External Form and Internal Structure.

[B52] Paffenhöfer G. A. (1998). On the relation of structure, perception and activity in marine planktonic copepods. J. Mar. Syst. 15, 457–473. 10.1016/S0924-7963(97)00037-7

[B53] Perales-Raya C. Almansa E. Bartolomé A. Felipe B. C. Iglesias J. Sánchez F. J. (2014). Age validation in *Octopus vulgaris* beaks across the full ontogenetic range: beaks as recorders of life events in octopuses. J. Shellfish Res. 33, 481–493. 10.2983/035.033.0217

[B54] Rose M. (1933). Copepodes pelagiques. Faune de France. Paris: Lachevalier.

[B55] Roura Á. Antón Álvarez-Salgado X. González Á. F. Gregori M. Rosón G. Otero J. (2016). Life strategies of cephalopod paralarvae in a coastal upwelling system (NW Iberian Peninsula): insights from zooplankton community and spatio-temporal analyses. Fish. Oceanogr. 25, 241–258. 10.1111/fog.12151

[B56] Roura A. Álvarez-Salgado X. A. González A. F. Gregori M. Rosón G. Guerra A. (2013). Short-term meso-scale variability of mesozooplankton communities in a coastal upwelling system (NW Spain). Prog. Oceanogr. 109, 18–32. 10.1016/j.pocean.2012.09.003

[B57] Roura Á. González Á. F. Redd K. Guerra Á. (2012). Molecular prey identification in wild *Octopus vulgaris* paralarvae. Mar. Biol. 159, 1335–1345. 10.1007/s00227-012-1914-9

[B58] Schoener T. W. (1971). Theory of feeding strategies. Annu. Rev. Ecol. Evol. Syst. 2, 369–404. 10.1146/annurev.es.02.110171.002101

[B59] Semmens J. M. Pecl G. T. Villanueva R. Jouffre D. Sobrino I. Wood J. B. (2004). Understanding octopus growth: patterns, variability and physiology. Mar. Freshw. Res. 55, 367–377. 10.1071/MF03155

[B60] Trégouboff G. Rose M. (1957). Acantharia, Manuel de Planctonologie Méditerranéenne. Paris: Paris Centre National de la Recherche Scientifique.

[B61] Valverde J. C. García B. G. (2005). Suitable dissolved oxygen levels for common octopus (*Octopus vulgaris* Cuvier, 1797) at different weights and temperatures: analysis of respiratory behaviour. Aquaculture 244, 303–314. 10.1016/j.aquaculture.2004.09.036

[B62] Vidal E. A. G. Villanueva R. Andrade J. P. Gleadail I. G. Iglesias J.. (2014). Cephalopod culture: current status of main biological models and research priorities. Adv. Cephalopod Sci. 67, 1–98. 10.1016/B978-0-12-800287-2.00001-9 24880794

[B63] Villanueva R. (1994). Decapod crab zoeae as food for rearing cephalopod paralarvae. Aquaculture 128, 143–152. 10.1016/0044-8486(94)90109-0

[B64] Villanueva R. (1995). Experimental rearing and growth of planktonic *Octopus vulgaris* from hatching to settlement. Can. J. Fish. Aquat. Sci. 52, 2639–2650. 10.1139/f95-853

[B65] Villanueva R. Norman M. D. (2008). Biology of the planktonic stages of benthic octopuses. Oceanogr. Mar. Biol. Annu. Rev. 46, 105–202. 10.1201/9781420065756.ch4

[B66] Villanueva R. Nozais C. Boletzky S. V. (1996). Swimming behaviour and food searching in planktonic *Octopus vulgaris* Cuvier from hatching to settlement. J. Exp. Mar. Biol. Ecol. 208, 169–184. 10.1016/S0022-0981(96)02670-6

[B67] Volkoff H. (2016). The neuroendocrine regulation of food intake in fish: a review of current knowledge. Front. Neurosci. 10:540. 10.3389/fnins.2016.00540 27965528 PMC5126056

[B68] Wells M. J. (1978). Octopus: Physiology and Behaviour of an Advanced Invertebrate. London: Chapman and Hall, Ltd.

[B69] Wells M. J. Wells J. (1985). Ventilation frequencies and stroke volumes in acute hypoxia in *Octopus*. J. Exp. Biol. 118, 445–448.

[B70] Westermann B. Ruth P. Litzlbauer H. D. Beck I. Beuerlein K. Schmidtberg H.. (2002). The digestive tract of *Nautilus pompilius* (Cephalopoda, Tetrabranchiata): an X-ray analytical and computational tomography study on the living animal. J. Exp. Biol. 205, 1617–1624. 12000806 10.1242/jeb.205.11.1617

[B71] Westermann B. Schipp R. (1998). Morphology and histology of the digestive tract of *Nautilus pompilius* and *Nautilus macromphalus* (Cephalopoda, Tetrabranchiata). Zoomorphology 117, 237–245. 10.1007/s004350050048

[B72] Wood J. D. (1969). Electrophysiological and pharmacological properties of the stomach of the squid *Loligo pealii* (Lesueur). Comp. Biochem. Physiol. 30, 813–824. 10.1016/0010-406X(69)90036-X 4390631

[B73] Yen J. Lenz P. H. Gassie D. V. Hartline D. K. (1992). Mechanoreception in marine copepods: electrophysiological studies on the first antennae. J. Plankton Res. 14, 495–512. 10.1093/plankt/14.4.495

[B74] Young J. Z. (1960). Unit processes in the formation of representations in the memory of octopus. Proc. R. Soc. Lond. B Biol. Sci. 153, 1–17. 10.1098/rspb.1960.0084

[B75] Young J. Z. (1965). The nervous pathways for poisoning, eating and learning in *Octopus*. J. Exp. Biol. 43, 581–593. 5863760 10.1242/jeb.43.3.581

[B76] Young J. Z. (1967). The visceral nerves of octopus. Philos. Trans. R. Soc. Lond. B Biol. Sci. 253, 1–22. 10.1098/rstb.1967.0032

[B77] Young J. Z. (1971). The Anatomy of the Nervous System of Octopus vulgaris. London, UK: Clarendon Press; Oxford University Press.

[B78] Zar J. H. (1999). Biostatistical Analysis, 4th Edition. ed Ryu T. Upper Saddle River, NJ: Prentice-Hall Inc.

