# Supplementary material for: Prey Capture, Ingestion, and Digestion Dynamics of Octopus vulgaris Paralarvae Fed Live Zooplankton
Source: Front Physiol. 2017 Aug 17;8:573. doi: 10.3389/fphys.2017.00573 (PMC5562678; doi:10.3389/fphys.2017.00573)
Supplement: Supplementary file 1 [file Table1.DOCX]

Supplementary material

Table 1. Mean and standard deviation of digestion parameters per prey (n) as measured on video recordings of effective attacks. Mantle contraction frequency (MC), buccal massmovement (BM), andradula movement frequency (RM), during the initial phase (IP), the middle phase (MP) and late phase (LP) of paralarvae-prey interaction. Total prey is the average value of the variables among the three interaction phases. The average No. of siphon propulsions (SP) is given for the whole ingestion period. See text for definitions.

| **Prey type** | **n** | **MC / 10 s** | | | | **BM / 10 s** | | | | **Radula movements / 10 s** | | | | **SP / 10 s** |
| --- | --- | --- | --- | --- | --- | --- | --- | --- | --- | --- | --- | --- | --- | --- |
|  |  | **IP** | **MP** | **LP** | **Total prey** | **IP** | **MP** | **LP** | **Total prey** | **IP** | **MP** | **LP** | **Total prey** | **Total prey** |
| *Acartia clausii* | 3 | 15.34 ± 0.58 | 13.67 ± 0.58 | 11.67 ± 0.58 | 13.56 ± 0.51 | 4.34 ± 0.58 | 3.67 ± 0.58 | 2.666 ± 0.58 | 3.56 ± 0.51 | 4.67 ± 1.15 | 3 ± 1 | 3 ± 0 | 3.56 ± 0.69 | 2.47 ± 0.57 |
| *Temora longicornis* | 4 | 14.50 ± 1.29 | 13.5 ± 1.29 | 11.5 ± 0.58 | 13.17 ± 1.04 | 7 ± 0.82 | 5.5 ± 1.3 | 5 ± 0.82 | 5.84 ± 0.79 | 5 ± 0.82 | 4.25 ± 0.96 | 4.25 ± 1.5 | 4.5 ± 1 | 3.13 ± 0.34 |
| *Centropages sp* | 3 | 17.00 ± 1.00 | 14.34 ± 2.08 | 13.34 ± 1.53 | 15.23 ± 0.19 | 4.67 ± 1.53 | 3.67 ± 0.58 | 3 ± 0 | 3.78 ± 0.69 | 4 ± 1 | 4 ± 1 | 5 ± 0 | 4.34 ± 0.33 | 2.47 ± 0.34 |
| *Podon intermedius* | 4 | 14.60 ± 1.52 | 13 ± 1 | 11.6 ± 0.9 | 13.27 ± 1.14 | 7.2 ± 1.48 | 6 ± 0.71 | 5.2 ± 0.84 | 6.54 ± 0.51 | 3.6 ± 0.89 | 5.8 ± 1.1 | 4 ± 1.23 | 4.47 ± 0.51 | 2.67 ± 0.557 |
| *Carcinus maenas zoeae* | 5 | 17.80 ± 0.84 | 13 ± 1 | 12.2 ± 1.30 | 14.34 ± 1.04 | 10.6 ± 1.14 | 9.6 ± 1.34 | 8 ± 0.71 | 9.4 ± 0.98 | 7.6 ± 1.14 | 6.4 ± 1.34 | 6.4 ± 1.14 | 6.8 ± 1.09 | 3.47 ± 0.57 |
| *Maja brachydactyla zoeae* | 5 | 17.60 ± 0.89 | 12.8 ± 0.84 | 11.8 ± 0.84 | 14.06 ± 0.19 | 12.8 ± 0.84 | 10.6 ± 1.14 | 8.4 ± 1.53 | 10.14 ± 1.02 | 8 ± 0.71 | 7 ± 0.71 | 6.4 ± 1.14 | 7.13 ± 0.77 | 3.34 ± 0.34 |
| *Cancer pagurus Zoeae* | 5 | 17.80 ± 0.84 | 13 ± 1 | 12.2 ± 1.3 | 14.34 ± 1.14 | 10.6 ± 1.14 | 9.6 ± 1.34 | 8 ± 0.71 | 9.4 ± 0.98 | 7.6 ± 1.14 | 6.4 ± 1.34 | 6.4 ± 1.14 | 8.3 ± 0.93 | 2.47 ± 0.57 |
| *Pisidia longicornis* | 3 | 16.00 ± 2.00 | 11.67 ± 0.58 | 11.67 ± 2.31 | 13.11 ± 0.75 | 8.67 ± 0.58 | 8 ± 1 | 8.333 ± 1.16 | 8.34 ± 0.34 | 5.67 ± 0.58 | 6.33 ± 1.53 | 6.33 ± 0.58 | 6.1 ± 0.76 | 3 ± 1 |
| *Paguridae* | 5 | 17.00 ± 0.71 | 14.4 ± 0.9 | 12.2 ± 0.84 | 14.54 ± 2.78 | 13.2 ± 2.17 | 11 ± 1.41 | 9.4 ± 0.55 | 11.34 ± 1.37 | 8.4 ± 0.55 | 6.8 ± 0.84 | 6.6 ± 0.89 | 7.27 ± 0.55 | 3.34 ± 0.43 |
| *Processidae* | 5 | 17.60 ± 0.55 | 13.8 ± 0.45 | 12.6 ± 0.54 | 14.34 ± 0.53 | 9.4 ± 1.14 | 8.6 ± 0.89 | 8.6 ± 1.34 | 8.87 ± 0.90 | 7.4 ± 1.34 | 6.2 ± 1.30 | 6.8 ± 0.84 | 6.8 ± 0.93 | 3.46 ± 0.57 |
| *Hippolytidae* | 5 | 17.40 ± 0.55 | 13.6 ± 1.67 | 13.6 ± 2.40 | 14.87 ± 0.77 | 8.4 ± 1.52 | 8.8 ± 1.48 | 8.4 ± 1.14 | 8.54 ± 0.38 | 7 ± 0.71 | 6.2 ± 1.30 | 7.2 ± 1.1 | 6.8 ± 0.77 | 2.6 ± 0.36 |
| *Palaemonidae* | 5 | 17.60 ± 0.55 | 14.4 ± 0.55 | 13.6 ± 2.08 | 15.2 ± 0.56 | 8.8 ± 1.3 | 8.4 ± 1.34 | 7 ± 1 | 8.07 ± 0.72 | 6.4 ± 0.89 | 7.2 ± 0.84 | 7.6 ± 1.14 | 7.07 ± 0.37 | 3.73 ± 0.36 |
| *Euphausiid* | 3 | 17.33 ± 0.58 | 13 ± 1.73 | 11.33 ± 0.58 | 13.89 ± 0.51 | 9 ± 0 | 8 ± 0 | 8 ± 1 | 8.34 ± 0.34 | 8.33 ± 0.58 | 7.33 ± 0.58 | 6.33 ± 0.58 | 7.34 ± 0.23 | 3.33 ± 0.34 |
