# Supplementary material for: Prey Capture, Ingestion, and Digestion Dynamics of Octopus vulgaris Paralarvae Fed Live Zooplankton
Source: Front Physiol. 2017 Aug 17;8:573. doi: 10.3389/fphys.2017.00573 (PMC5562678; doi:10.3389/fphys.2017.00573)
Supplement: Supplementary file 2 [file Table2.DOCX]

Supplementary material

Table 2. Mean and standard deviation of digestion parameters per prey (n) as measured on video recordings of effective attacks Terminal intestine contraction frequency (TIC) during the initial phase (IP), the middle phase (MP) and the late phase (LP) of paralarvae-prey interaction. The frequency of stomach contraction (SC), and peristaltic crop movement (PC)are shown together with total ingestion time (Ti) and total contact time (TCT) in seconds. Total prey is the average value of the variables among the three interaction phases See text for definitions.

| **Prey type** | **n** | **TIC / 10 s** | | | | **SC / 10 s** | **PC / 10 s** | **Ti (s)** | **TCT (s)** |
| --- | --- | --- | --- | --- | --- | --- | --- | --- | --- |
|  |  | **IP** | **MP** | **LP** | **Total prey** | **Mean all phases** | **Mean all phases** | **Mean all phases** | **Mean all phases** |
| *Acartia clausii* | 3 | nd | nd | nd | nd | 4.34 ± 0.67 | nd | 35 ± 8.54 | 49.67 ± 8.33 |
| *Temora longicornis* | 4 | 8.00 ± 0.82 | 7.5 ± 1.29 | 6 ± 0.82 | 7.17 ± 0.58 | 5 ± 1 | nd | 80.5 ± 12.26 | 92.75 ± 13.69 |
| *Centropages sp* | 3 | 7.67 ± 0.58 | 7 ± 1 | 7.34 ± 1.53 | 7.34 ± 0.88 | 5.34 ± 1.29 | nd | 87 ± 5.57 | 98.67 ± 2.08 |
| *Podon intermedius* | 4 | 7.00 ± 0.71 | 6.8 ± 0.05 | 8.2 ± 0.45 | 7.34 ± 0.24 | 4.2 ± 0.67 | 2.47 ± 0.56 | 81.8 ± 18.92 | 91 ± 17.72 |
| *Carcinus maenas zoeae* | 5 | 7.80 ± 0.84 | 6.6 ± 0.55 | 7.6 ± 0.55 | 7.17 ± 0.58 | 5.6 ± 0.55 | 2.47 ± 0.56 | 171.4 ± 20.44 | 216.4 ± 17.97 |
| *Maja brachydactyla zoeae* | 5 | 7.80 ± 0.45 | 6.6 ± 0.5 | 6.2 ± 0.84 | 6.87 ± 0.38 | 5.2 ± 0.37 | 3.13 ± 0.3 | 175 ± 9.82 | 230.6 ± 17.9 |
| *Cancer pagurus zoeae* | 5 | 8.20 ± 0.45 | 7.2 ± 0.45 | 6.6 ± 0.55 | 7.6 ± 0.24 | 5.3 ± 0.67 | 2.47 ± 0.56 | 171.4 ± 20.44 | 225.6 ± 19.46 |
| *Pisidia longicornis* | 3 | 7.67 ± 0.58 | 6 ± 0 | 6.34 ± 0.58 | 6.67 ± 0.34 | 5.67 ± 0.57 | 2.01 ± 1.02 | 173 ± 14.93 | 232.67 ± 28.53 |
| *Paguridae* | 5 | 8.20 ± 0.45 | 7 ± 0 | 6.8 ± 0.84 | 7.34 ± 0.24 | 5.3 ± 0.87 | 2.94 ± 0.43 | 129.6 ± 25.62 | 206.4 ± 29.36 |
| *Processidae* | 5 | 7.80 ± 0.45 | 7.2 ± 1.1 | 7 ± 0.71 | 7.34 ± 0.34 | 5 ± 1 | 2.46 ± 0.56 | 155.4 ± 34.28 | 231.8 ± 29.15 |
| *Hippolytidae* | 5 | 7.20 ± 0.45 | 7.2 ± 0.37 | 7.8 ± 1.09 | 7.4 ± 0.44 | 5.3 ± 1.11 | 2.6 ± 0.37 | 135.6 ± 26.75 | 207.6 ± 23.86 |
| *Palaemonidae* | 5 | 8.40 ± 1.34 | 7.2 ± 0.45 | 7.6 ± 0.55 | 7.73 ± 0.28 | 5.6 ± 0.55 | 2.73 ± 0.36 | 133.4 ± 32.42 | 217.6 ± 42.36 |
| *Euphausiid* | 3 | 8.33 ± 1.15 | 7.34 ± 0.58 | 6.67 ± 1.15 | 7.45 ± 0.19 | 5.6 ± 1.15 | 2.33 ± 0.34 | 124.66 ± 14.01 | 216.33 ± 8.39 |
