# Supplementary material for: Prey Capture, Ingestion, and Digestion Dynamics of Octopus vulgaris Paralarvae Fed Live Zooplankton
Source: Front Physiol. 2017 Aug 17;8:573. doi: 10.3389/fphys.2017.00573 (PMC5562678; doi:10.3389/fphys.2017.00573)
Supplement: Supplementary file 3 [file Table3.DOCX]

Supplementary material

Table 3. Mean and standard deviation of digestion parameters per prey (n) as measured on video recordings of effective attacks. TS, time spanned between prey capture and food reaching the stomach; Sv, stomach volume; Cv, crop volume; TF, total food volume; IR, Ingestion rate. See text for definitions.

| **Prey type** | **n** | **TS(s)** | **Sv (mm^3^)** | **Cv (mm^3^)** | **TF (mm^3^)** | **IR (mm^3^/min)** |
| --- | --- | --- | --- | --- | --- | --- |
| *Acartia clausii* | 3 | 14.67 ± 03.21 | 0.003 ± 0.002 | 0.004 ± 0.001 | 0.007 ± 0.003 | 0.008 ± 0.002 |
| *Temora longicornis* | 4 | 12.25 ± 01.71 | 0.006 ± 0.002 | 0.006 ± 0.001 | 0.013 ± 0.002 | 0.009 ± 0.002 |
| *Centropages sp* | 3 | 11.67 ± 04.04 | 0.008 ± 0.002 | 0.009 ± 0.001 | 0.017 ± 0.005 | 0.012 ± 0.003 |
| *Podon intermedius* | 4 | 9.20 ± 05.07 | 0.002 ± 0.01 | 0.003 ± 0.001 | 0.012 ± 0.01 | 0.009 ± 0.002 |
| *Carcinus maenas zoeae* | 5 | 45.00 ± 05.66 | 0.015 ± 0.008 | 0.09 ± 0.01 | 0.116 ± 0.02 | 0.041 ± 0.007 |
| *Maja brachydactyla zoeae* | 5 | 55.60 ± 08.74 | 0.014 ± 0.006 | 0.081 ± 0.01 | 0.095 ± 0.02 | 0.043 ± 0.013 |
| *Cancer pagurus zoeae* | 5 | 54.20 ± 07.12 | 0.015 ± 0.004 | 0.1 ± 0.01 | 0.115 ± 0.05 | 0.041 ± 0.012 |
| *Pisidia longicornis* | 3 | 59.67 ± 24.34 | 0.013 ± 0.007 | 0.11 ± 0.02 | 0.117 ± 0.05 | 0.033 ± 0.013 |
| *Paguridae* | 5 | 76.80 ± 07.26 | 0.014 ± 0.008 | 0.088 ± 0.05 | 0.117 ± 0.63 | 0.054 ± 0.029 |
| *Processidae* | 5 | 76.40 ± 08.96 | 0.016 ± 0.007 | 0.1 ± 0.02 | 0.121 ± 0.036 | 0.046 ± 0.011 |
| *Hippolytidae* | 5 | 72.00 ± 05.15 | 0.016 ± 0.009 | 0.085 ± 0.04 | 0.101 ± 0.042 | 0.044 ± 0.02 |
| *Palaemonidae* | 5 | 84.20 ± 14.38 | 0.017 ± 0.008 | 0.098 ± 0.06 | 0.115 ± 0.061 | 0.051 ± 0.012 |
| *Euphausiid* | 3 | 91.66 ± 10.59 | 0.018 ± 0.008 | 0.098 ± 0.05 | 0.117 ± 0.096 | 0.056 ± 0.018 |
