# Supplementary material for: Prey Capture, Ingestion, and Digestion Dynamics of Octopus vulgaris Paralarvae Fed Live Zooplankton
Source: Front Physiol. 2017 Aug 17;8:573. doi: 10.3389/fphys.2017.00573 (PMC5562678; doi:10.3389/fphys.2017.00573)
Supplement: Supplementary file 4 [file Table4.DOCX]

Supplementary material

Table 4. Mean and standard deviation of paralarvae parameters per prey (n) as measured on video recordings of ineffective attacks. MC, mantle contraction; BM buccal mass movement; RM, radula movement, during the initial phase (IP), the middle phase (MP) and the late phase (LP) a of paralarvae-prey interaction. Total prey is the average value of the variables among the three interaction phases

| **Prey type** | **n** | **(MC / 10 s)** | | | | **(BM / 10 s)** | | | | **T (TIC / 10 s)** | | | | **Total time (s)** |
| --- | --- | --- | --- | --- | --- | --- | --- | --- | --- | --- | --- | --- | --- | --- |
|  |  | **IP** | **MP** | **LP** | **Total prey** | **IP** | **MP** | **LP** | **Total prey** | **IP** | **MP** | **LP** | **Total prey** |  |
| *Crangonidae* | 3 | 17.334 ± 0.577 | 18.34 ± 0.58 | 17 ± 2 | 17.56 ± 0.38 | 7.67 ± 0.58 | 7.34 ± 1.53 | 7 ± 2.65 | 7.34 ± 1.45 | 5.34 ± 0.58 | 5 ± 0 | 5.34 ± 1.15 | 5.23 ± 0.19 | 44.34 ± 7.64 |
| *Pisidia longicornis* | 2 | 18.334 ± 0.577 | 18.34 ± 0.58 | 17.67 ± 0.58 | 19.12 ± 0.51 | 10 ± 1.73 | 10 ± 2.65 | 9.67 ± 1.16 | 9.89 ± 1.58 | 5.67 ± 1.53 | 4.67 ± 1.16 | 4 ± 1 | 5 ± 0.47 | 107 ± 59.10 |
| *Paguridae* | 4 | 16.750 ± 2.217 | 17.75 ± 0.96 | 18.75 ± 1.89 | 18 ± 0.58 | 11 ± 0.82 | 9.75 ± 1.71 | 10.25 ± 0.96 | 10.34 ± 1 | 4.5 ± 1 | 3.75 ± 0.96 | 5 ± 0.82 | 4.5 ± 0.24 | 55 ± 14.72 |
| *Processidae* | 4 | 16.250 ± 1.500 | 16.5 ± 1.29 | 18 ± 1.15 | 16.56 ± 0.19 | 9.75 ± 0.96 | 9.75 ± 1.26 | 10.25 ± 0.96 | 10 ± 0.34 | 4.75 ± 0.5 | 4 ± 1.41 | 5 ± 0.82 | 4.45 ± 0.19 | 60.5 ± 16.66 |
| *Hippolytidae* | 4 | 18.500 ± 0.577 | 17.75 ± 0.96 | 18.25 ± 1.5 | 18.12 ± 0.39 | 10 ± 0.82 | 9.25 ± 0.5 | 10.25 ± 2.06 | 9.78 ± 0.7 | 6 ± 1.41 | 4.75 ± 0.96 | 6.25 ± 0.5 | 5.67 ± 1 | 63 ± 24.26 |
| *Palaemonidae* | 4 | 18.500 ± 0.577 | 17.75 ± 0.96 | 18.25 ± 1.5 | 18.12 ± 0.39 | 10 ± 0.2 | 9.25 ± 0.5 | 10.65 ± 1.55 | 9.77 ± 0.7 | 5 ± 0 | 4.75 ± 0.96 | 5.25 ± 0.5 | 4.89 ± 0.38 | 59.5 ± 16.82 |
| Gastropods | 3 | 18.667 ± 0.577 | 17.67 ± 1.15 | 18 ± 1.73 | 18.12 ± 0.39 | 11.67 ± 2.08 | 10.67 ± 2.08 | 11 ± 1.73 | 11.12 ± 0.96 | 4.67 ± 0.58 | 5 ± 1 | 6 ± 1 | 5.22 ± 0.83 | 35.34 ± 8.15 |
| *Maja brachydactyla zoea* | 3 | 16.667 ± 0.577 | 16.34 ± 1.53 | 16.34 ± 1.15 | 16.45 ± 0.77 | 12.67 ± 0.58 | 14.34 ± 3.51 | 13 ± 1.73 | 13.34 ± 1.45 | 4.5 ± 0.71 | 5 ± 1.414 | 6 ± 0 | 5.23 ± 0.51 | 69.34 ± 21.78 |
| Hyperiid amphipod | 3 | 17.000 ± 1.000 | 14.67 ± 1.16 | 16.34 ± 1.53 | 16 ± 0.88 | 16 ± 1 | 14.34 ± 1.16 | 16 ± 2.65 | 15.45 ± 0.96 | nd | nd | nd | nd | 128.34 ± 71.56 |
| Gammaridae | 2 | 17.000 ± 1.414 | 14 ± 1.41 | 17 ± 0 | 16 ± 0 | 13.5 ± 0.71 | 15.5 ± 0.71 | 13 ± 1.41 | 14 ± 0.94 | nd | nd | nd | 4.17 ± 0.24 | 153 ± 19.79 |
| Brachyura megalopa | 2 | 15.000 ± 1.414 | 15.5 ± 2.12 | 13.5 ± 0.71 | 14.6 ± 1.02 | 13 ± 2.83 | 14 ± 0 | 14.5 ± 0.71 | 13.83 ± 0.71 | 4.5 ± 0.71 | 4 ± 0 | 4 ± 1.41 | nd | 55.5 ± 13.44 |
